# A Stochastic Landscape Approach for Protein Folding State Classification

**DOI:** 10.1021/acs.jctc.4c00464

**Published:** 2024-06-26

**Authors:** Michael Faran, Dhiman Ray, Shubhadeep Nag, Umberto Raucci, Michele Parrinello, Gili Bisker

**Affiliations:** † Department of Biomedical Engineering, Faculty of Engineering, Tel Aviv University, Tel Aviv 69978, Israel; ‡ Atomistic Simulations, Italian Institute of Technology, Via Enrico Melen 83, 16152 Genova, Italy; ¶ The Center for Physics and Chemistry of Living Systems, Tel Aviv University, Tel Aviv 6997801, Israel; § The Center for Nanoscience and Nanotechnology, Tel Aviv University, Tel Aviv 6997801, Israel; ∥ The Center for Light-Matter Interaction, Tel Aviv University, Tel Aviv 6997801, Israel

## Abstract

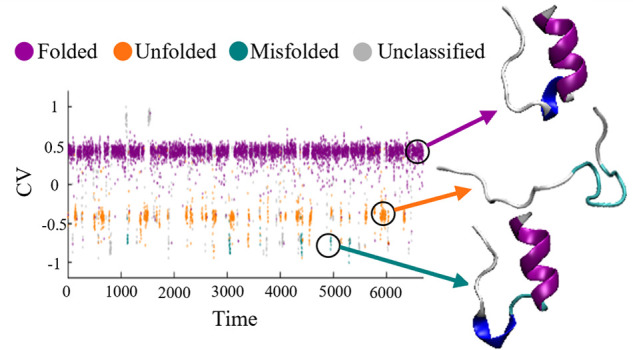

Protein folding is a critical process that determines the functional state of proteins. Proper folding is essential for proteins to acquire their functional three-dimensional structures and execute their biological role, whereas misfolded proteins can lead to various diseases, including neurodegenerative disorders like Alzheimer’s and Parkinson’s. Therefore, a deeper understanding of protein folding is vital for understanding disease mechanisms and developing therapeutic strategies. This study introduces the Stochastic Landscape Classification (SLC), an innovative, automated, nonlearning algorithm that quantitatively analyzes protein folding dynamics. Focusing on collective variables (CVs) – low-dimensional representations of complex dynamical systems like molecular dynamics (MD) of macromolecules – the SLC approach segments the CVs into distinct macrostates, revealing the protein folding pathway explored by MD simulations. The segmentation is achieved by analyzing changes in CV trends and clustering these segments using a standard density-based spatial clustering of applications with noise (DBSCAN) scheme. Applied to the MD-based CV trajectories of Chignolin and Trp-Cage proteins, the SLC demonstrates apposite accuracy, validated by comparing standard classification metrics against ground-truth data. These metrics affirm the efficacy of the SLC in capturing intricate protein dynamics and offer a method to evaluate and select the most informative CVs. The practical application of this technique lies in its ability to provide a detailed, quantitative description of protein folding processes, with significant implications for understanding and manipulating protein behavior in industrial and pharmaceutical contexts.

## 1 Introduction

Proteins are biological macromolecules that play critical roles in almost all physiological processes in living organisms. The majority of known proteins form well-defined three-dimensional folded structures that dictate their biological functions. The phenomenon of protein folding, i.e., a conformational transition between a disordered polymer of constituent amino acids and the folded native conformation, attracted significant interest over the past century.^1−8^ Furthermore, protein misfolding has been associated with several adverse medical conditions, including Alzheimer’s disease, Bovine spongiform encephalopathy, and sickle cell anemia.^9−11^ The seminal work of Anfinsen in the early 1960*s* marked the beginning of significant advancements in protein folding studies, propelled by a synergistic blend of experimental and theoretical approaches.^12−15^ A variety of experimental techniques, such as X-ray crystallography, NMR spectroscopy, fluorescence spectroscopy, and cryo-EM, have played a pivotal role in studying the protein folding problem.^16−18^ The most recent paradigm shift in the field came from the AlphaFold2 deep-learning model which could predict folded structures from amino acid sequences with unprecedented accuracy.^19^ In addition to structural biology experiments and data-driven algorithms, statistical-mechanics-based approaches, particularly molecular dynamics (MD) simulations, played a fundamental role in uncovering the atomistic details of the protein folding mechanism.^20−26^ One way to interpret the protein folding mechanism from MD simulations is by clustering the sampled configurations into different macrostates. Particularly notable are k-means clustering,^27^ gromos clustering,^28^ and t-distributed stochastic neighbor embedding (t-SNE).^29^ Such clustering is often performed in high dimensional coordinate space and does not include any kinetic information which is vital for deciphering the correct folding mechanism. Due to the overall complexity of the protein folding process, which involves several intramolecular and intermolecular interactions, it is often convenient to describe the key dynamics modes of the protein in terms of a reduced dimensional representation. Such a representation is referred to as a collective variable (CV) which is a function of the 3*N* dimensional coordinates of the protein atoms and is designed to encode the slow modes of the system.^30^ Many CVs have been historically used for the study of protein folding, including root-mean-square deviation (RMSD),^31^ the radius of gyration (*R*_*g*_),^32^ and end-to-end distance.^33^ However, these intuitive CVs often fail to capture the true complexity of the protein folding problem.^34,35^ To address this problem, machine learning, generally used for biological systems,^36,37^ can combine several molecular descriptors into a low-dimensional space to capture the intricate details of protein folding dynamics. Methods in this category include principal component analysis (PCA),^38^ time-lagged independent component analysis (TICA),^39^ harmonic linear discriminant analysis (HLDA),^40,41^ deep linear discriminant analysis (Deep-LDA),^42,43^ deep time-lagged independent component analysis (Deep-TICA),^44^ and deep targeted discriminant analysis (Deep-TDA).^45,46^ In a pioneering study, Bhowmik et al. succeeded in clustering protein conformations with 89% accuracy using a deep learning algorithm called convolutional variational autoencoder (CVAE).^47^ However, their approach did not consider any kinetic information in the clustering process. Taking into account the kinetic information is challenging, as protein folding is a rare event where the associated metastable states (i.e., folded, unfolded, and intermediate states) are separated by free energy barriers. Hence, the transitions between these states are stochastic. This stochasticity arises from the thermal fluctuations resulting from the interaction of the protein with the solvent and other molecular crowders at a physiological temperature.^48−50^ Albeit stochastic, metastable transition statistics is well characterized,^51^ and can be detected using a data-driven approach of combining a Koopman-based model with the tensor train format.^52^ Thus, this prior information can be potentially exploited for metastable state classification along a CV.

The BEAST (Bayesian Estimator of Abrupt change, Seasonality, and Trend)^53^ is an algorithm that automatically searches for trend-change points in stochastic trajectories,^54^ which can be advantageous for data classification. When the protein is found in either metastable basins, it fluctuates around the same physical values in the free energy landscape. A trend, on the other hand, is a statistically significant monotonic change in a variable over time.^55,56^ Therefore, from an observable trajectory perspective, the trend around metastable states is expected to be zero, and a nonzero trend would characterize the transition between these basins. As a result, it becomes feasible to automatically find trajectory segments assosiated with a metastable state by detecting trend changes. This approach has been recently demonstrated for predicting self-assembly times in a toy physical model of interacting particles under nonequilibrium conditions.^57^

Here, we present a technique that exploits the protein folding stochasticity to automatically detect and classify metastable states in a CV time series. The only two requirements are that the protein visits the associated metastable states along the trajectory multiple times and that its states are discernible by the analyzed observable. We name this procedure the Stochastic Landscape Classification (SLC). We apply the SLC to identify the metastable states associated with folding two model proteins, namely, Chignolin and Trp-cage. Their relatively simple structures and microsecond time scale folding behavior^58,59^ make them ideal candidates for testing new algorithms for performing or analyzing molecular simulations. As the quality of MD simulation and analysis depends critically on the quality of the chosen CV, we compare the ability of several intuitive and machine-learning CVs to identify the metastable states of a protein by the SLC. We show that, when used in combination with an optimal CV trained as a function of system-agnostic feature space, the SLC can cluster the different protein conformations with almost 90% accuracy. Our approach can be generalized to detect and classify other rare biomolecular events, such as protein–ligand binding and RNA conformational dynamics. The SLC can be further implemented on time-series data from experimental measurements, like single-molecule force spectroscopy and fluorescence correlation spectroscopy.

## 2 Methods

### 2.1 The Stochastic Landscape Classification

The Stochastic Landscape Classification (SLC) is an empirical approach to detect and classify protein states along a CV trajectory.^57^ The key idea is to segment the trajectory data using the BEAST algorithm and exploit the statistical information on the segments as input for the unsupervised classification of the protein states. Unlike the Stochastic Landscape Method, developed for self-assembly inference,^57^ the proposed technique does not rely on learning from previous data. A prerequisite is that the protein visits each relevant metastable state along the path a few times. Moreover, the underlying assumption is that the protein spends much of its time in metastable states, with a short transition periods between them, following the “low rattling” concept.^60^ We apply our approach to CV exemplary trajectories provided for the Chignolin and Trp Cage MD simulations, eventually compared and evaluated to ground truth values obtained from multiple microseconds of unbiased molecular dynamics trajectories. The CV trajectories, including the ground truth classification, are taken from Shaw and co-workers.^61,62^ In these studies, classical MD simulations were used to model various proteins with the goal of understanding their folding mechanism by comparing the simulations with experimental results. Comprehensive details on implementing the stochastic landscape classification methodology can be found in our open-source documentation.^63^

#### 2.1.1 The BEAST Algorithm

The suggested states classification technique utilizes the BEAST algorithm as an automated method to divide a trajectory observable into segments with distinct statistical characteristics. The BEAST was chosen for this task because of its versatility, robustness, and ability to operate without prior knowledge of the number of statistical change points in the data. Instead, only an upper limit on the number of change points is required. The BEAST algorithm estimates the probability distribution of the number of statistical changes in the data and selects the median value of this distribution by default. This estimation is based on the assumption that a given time series consists of intervals of varying lengths, each with its Gaussian noise characteristics. The underlying unknown model is defined by a set of parameters, including the periods of seasonal signals, the number and timing of seasonal and trend change points, the parameters of a trend and an harmonic model of seasonality, and the magnitude of the Gaussian noise in each segment. The BEAST aims to identify the model parameters that generate the observed time series statistics using Bayesian inference. This involves maximizing the posterior probability distribution, which is the product of the likelihood function and the prior distribution of the model parameters, as per Bayes’ theorem. Since the posterior distribution cannot practically be calculated analytically, the algorithm employs a Monte Carlo Markov Chain to sample this distribution for posterior inference. While the BEAST can detect trend and seasonal change points, this study focuses exclusively on segmenting the data based on trend changes due to the absence of seasonality. A minimum duration for each segment was not required as a prerequisite. A quantitative perspective of the BEAST algorithm is given in Section S1 of the Supporting Information (SI).

#### 2.1.2 The SLC Flowchart

In the following, we outline the sequential steps of our stochastic landscape classification approach. First, the process is initiated by choosing a CV trajectory of interest as an input. Second, this CV trajectory may undergo down-sampling, if necessary, to optimize the computational run-time and efficiency of the BEAST algorithm. The third step involves directly applying the BEAST algorithm to the data, considering only trend changes, so the CV trajectory is divided into segments. Fourth, we focus on extracting stochastic coordinates^57^ from each segment of the data, including the mean, standard deviation (STD), and average trend. To ensure comparability across different segments, we standardize these coordinates by subtracting the mean value of each coordinate from all corresponding coordinates and then dividing the result by the standard deviation. This normalization process is applied simultaneously across all segments. Following normalization, we conduct a Principal Component Analysis (PCA) on the standardized stochastic coordinates, providing the final form of the stochastic coordinates suitable for subsequent analyses. We construct the “Stochastic Landscape” by representing each segment as a point corresponding to its respective stochastic coordinates in a three-dimensional scatter plot of the segment data. The sixth step involves the application of a clustering algorithm, namely the density-based spatial clustering of applications with noise (DBSCAN),^64^ with heuristically chosen parameters such as the radius of a neighborhood (ϵ) and the minimum cluster size (*minPts*) to ensure optimal clustering. A detailed description of DBSCAN classification is given in Section S2.1 of the SI.

In the seventh step, we compare the clusters identified by the DBSCAN algorithm to any available ground-truth data. It is important to note that the number of clusters generated by DBSCAN may differ from those in the ground-truth data. To address this, we employ the Kuhn-Munkres algorithm for label matching,^65,66^ ensuring a proper correlation between the identified clusters and specific protein states. Finally, we map the cluster labels back to their original segments in the CV trajectory. This allows us to label each segment appropriately, effectively dividing the CV trajectory into different states based on the analysis. The flowchart of our approach is depicted in Table 1.

**Table 1:** A Table Illustrating the Steps Involved in the Stochastic Landscape Classification^a^

Step	Description
1	Input: CV vs Time
2	Downsample (optional)
3	BEAST
4	Stochastic Coordinates Extraction
5	Stochastic Landscape Construction
6	DBSCAN Classification
7	Kuhn-Munkres Label Matching (optional)
8	Output: Protein States vs Time

a Steps 2 and 7 are optional and may not be applicable in all cases.

While our technique is implemented on protein folding as an example, its application is versatile and can be extended to classify observable trajectories in other biological systems that exhibit repeated transitions between specific metastable states.

#### 2.1.3 Collective Variables

In our analysis, we used some collective variables based on physical intuition, such as the root mean squared deviation (RMSD) and the end-to-end distance, due to their effectiveness in capturing the conformational changes of the proteins. These intuitive CVs provide a minimal description of protein conformational transitions and are often used when no further system-specific molecular level information is available. Additionally, we employed more advanced order parameters derived using neural networks.^42^ This approach enables the construction of highly efficient nonlinear CVs capable of distinguishing between multiple states. Among these CVs, the most important is the Deep Targeted Discriminant Analysis (Deep-TDA), which is used to define ground-truth data. Deep-TDA CVs are designed specifically to distinguish between metastable configurations of molecular systems. For example, if one trains a Deep-TDA to discriminate between states A and B, these two states would be distributed at specific locations in the CV space with a specific variance. Both the mean and variance are predefined by the user, making it possible to designate with certainty the configurations associated with each of the states A and B in the CV space. If transition path information between states is also available, an improved version of Deep-TDA, known as transition path informed Deep-TDA (TPI-Deep-TDA), could also be used to distinguish between the metastable states as well as the transition paths.

In Deep-TDA, physical descriptors (i.e., distances, dihedral angles) derived from unbiased molecular dynamics simulations confined within metastable basins are fed into a feed-forward neural network (NN). This NN is optimized according to a discrimination criterion. The goal is for the training data distribution to align with a predefined target distribution. Specifically, for a system with *N*_*S*_ metastable states, the NN is trained to map the multidimensional space of descriptors into a low-dimensional CV space, in which the distribution of training data matches a target distribution. A series of N_*S*_ Gaussians with predefined positions and widths are chosen as targets, and the neural network is trained to ensure that when the configurations from various metastable basins are projected onto the CV space, they are distributed according to the target Gaussians. In the case of a one-dimensional CV, there are two terms for each metastable state *k* in the loss function: one enforcing the mean value μ_*k*_ of its distribution in the CV-space, and the other its standard deviation σ_*k*_ to match the respective targets μ_*k*_^*tg*^ and σ_*k*_^*tg*^. Readers seeking further details are referred to ref (45).

### 2.2 Computational Details

In our study, we have analyzed molecular trajectories which sampled both folding and unfolding events for two distinct protein systems. These trajectories were generated in previous work^61,62^ using atomistic molecular dynamics simulations, leveraging classical force fields to model the intricate behaviors of atoms and molecules. We studied two protein systems, Chignolin and Trp-Cage. The former has only two free energy minima corresponding to the folded and the unfolded states, while the latter also has a third metastable state of a misfolded configuration. Therefore, studying these two proteins covers both the single-step and multistep protein folding dynamics. A brief description of these two proteins is presented below.

#### 2.2.1 Chignolin

Chignolin, a fast-folding peptide comprising 10 amino acid residues, is frequently employed as a model system for testing enhanced sampling algorithms.^67−72^ In our current analysis, we analyze an extensive 106 μs unbiased trajectory of Chignolin,^61^ generated using the CHARMM22* force field.^73^ Prior to applying the BEAST analysis, the trajectory was projected along five distinct one-dimensional CVs, including the end-to-end distance which measures the spatial distance between the *C*_α_ atoms of the first and tenth residues (CV _CH_^DIST^), RMSD of the *C*_α_ atoms (CV _CH_^RMSD^), Harmonic Linear Discriminant Analysis (HLDA)-based CV trained on selected interatomic contacts^41^ (CV _CH_^HLDA^), a deep targeted discriminant analysis (Deep-TDA) CV,^45^ trained on 45 pairwise contacts between the ten *C*_α_ atoms as the feature space,^46^ to distinguish the folded and unfolded states of Chignolin (CV _CH_^Deep-TDA^), and a transition path informed deep targeted discriminant analysis (TPI-Deep-TDA) CV,^46^ employing the same feature space as CV _CH_^Deep-TDA^, to distinguish the folded state, unfolded state, and the transition pathways sampled using on-the-fly probability enhanced sampling flooding (OPES_f_)^43^ simulations (CV _CH_^TPI-Deep-TDA^).

#### 2.2.2 Trp-Cage

Trp-Cage, a 20-residue protein known for its rapid folding, presents a more complex free energy landscape compared to Chignolin.^58^ It does not only have a folded and unfolded states, but also assumes a third, metastable misfolded state. In our study, we examine an extensive ∼268 μs unbiased trajectory of Trp-Cage, propagated using the DES-Amber force field field.^62^ For efficient computational processing with the BEAST algorithm, the trajectory was downsampled to 6692 frames, equally spaced over time. Prior to the BEAST analysis, the trajectory was projected onto nine one-dimensional CVs, including the slowest degree of freedom^44^ from a deep-TICA CV trained on the entire trajectory (CV _TRP_^Deep-TICA-I^), the second slowest degree of freedom obtained from the same deep-TICA CV of CV _TRP_^Deep-TICA-I^ (CV _TRP_^Deep-TICA-II^), a two-state Deep-TDA CV trained to distinguish the folded and unfolded states (CV _TRP_^Deep-TDA-I^), the first node of a three-state Deep-TDA CV trained to distinguish the folded, misfolded, and unfolded states (CV _TRP_^Deep-TDA-II^), the second node of the Deep-TDA CV from CV _TRP_^Deep-TDA-II^, trained to distinguish the metastable state from the folded and the unfolded states without distinguishing between the latter two (CV _TRP_^Deep-TDA-III^), the backbone RMSD of the Trp-Cage protein (CV _TRP_^RMSD-Backbone^), the end-to-end distance measured as the distance between the *C*_α_ atoms of first and twentieth residues (CV _TRP_^Dist^), the fraction of native contacts (CV _TRP_^Contact^), and the RMSD of all the heavy atoms (CV _TRP_^RMSD^).

For all the neural network based deep CVs, the feature space is a 190-dimensional vector composed of the pairwise contacts between all 20 *C*_α_ atoms. The training of the deep CVs is performed before the downsampling of the trajectory to increase the amount of training and test data sets.

## 3 Results and Discussion

### 3.1 Evaluation Metrics

The effectiveness of our clustering approach is rigorously assessed by comparing the results from the DBSCAN clustering algorithm to the established ground truth data for all the CVs (see Section S2.1 of the SI). To facilitate this comparison, we initially employ the Kuhn-Munkres algorithm, as detailed in Section S2.2 of the SI. Subsequently, we evaluate the state classification accuracy using a standard similarity metrics,^74^ including the Rand index (RI),^75^ the adjusted Rand index (ARI),^75^ the normalized mutual information index (NMI),^76^ the Dice index,^77^ the Jaccard index (JD),^78^ and the Fowlkes–Mallows index (FM).^79^ As these indices approach the value of 1, they suggest a closer alignment between the clustering results and the ground truth. Among the chosen indices, NMI exhibits nonlinear behavior in relation to the pair similarity true positive, where only high true positive values achieve NMI values closer to 1.^80^ For instance, 93% correct clustering might correspond to an NMI value of around 0.6. This set of indices offers a comprehensive evaluation of our clustering methodology, ensuring that our analysis is both robust and reliable.

### 3.2 Metastable State Identification for Chignolin

#### 3.2.1 Ground Truth Data

The CV _CH_^TPI-Deep-TDA^ values are considered as the ground-truth data in this case, since the TPI-Deep-TDA CV is explicitly designed to distinguish between the folded, unfolded, and transition state ensemble.^46^ In the space of CV _CH_^TPI-Deep-TDA^, the mean and width of the distribution of each of these states are predefined, where the width around the mean sets the boundaries for the CV values associated with these states. Therefore, projecting the trajectory along CV _CH_^TPI-Deep-TDA^ allows us to demarcate the different states of the Chignolin protein most accurately.

The classification of states is based on the CV _CH_^TPI-Deep-TDA^ values as follows. State 1, which corresponds to the folded state, is defined by CV _CH_^TPI-Deep-TDA^ values in the range [−7.671, – 1.954]. On the other hand, State 2, which corresponds to the unfolded state, includes CV _CH_^TPI-Deep-TDA^ values in the range [3.288,7.608]. For the intermediate range of [−1.954, 3.288], the classification of samples is ambiguous and not clearly defined as either folded or unfolded. For the purposes of establishing a baseline for comparison, a criterion has been arbitrarily established such that samples with a CV _CH_^TPI-Deep-TDA^ value less than 3.288 are classified as State 1 (folded), whereas samples with a CV _CH_^TPI-Deep-TDA^ value exceeding 3.288 are classified as State 2 (unfolded). This methodological approach facilitates a structured comparison for a ground truth.

After categorizing the states as described, we proceed to label each trajectory sample according to its identified state. This creates a time-series vector where each data point is annotated with its corresponding state label. Moving forward, this labeled vector serves as the benchmark or “ground truth” against which we compare and validate our subsequent analyses.

#### 3.2.2 The SLC Implementation

Implementing our approach involves processing all given CV trajectories as input, as outlined in Section 2.1.2. To illustrate, we detail the process for CV _CH_^TPI-Deep-TDA^ as an example.

**Figure 1:**
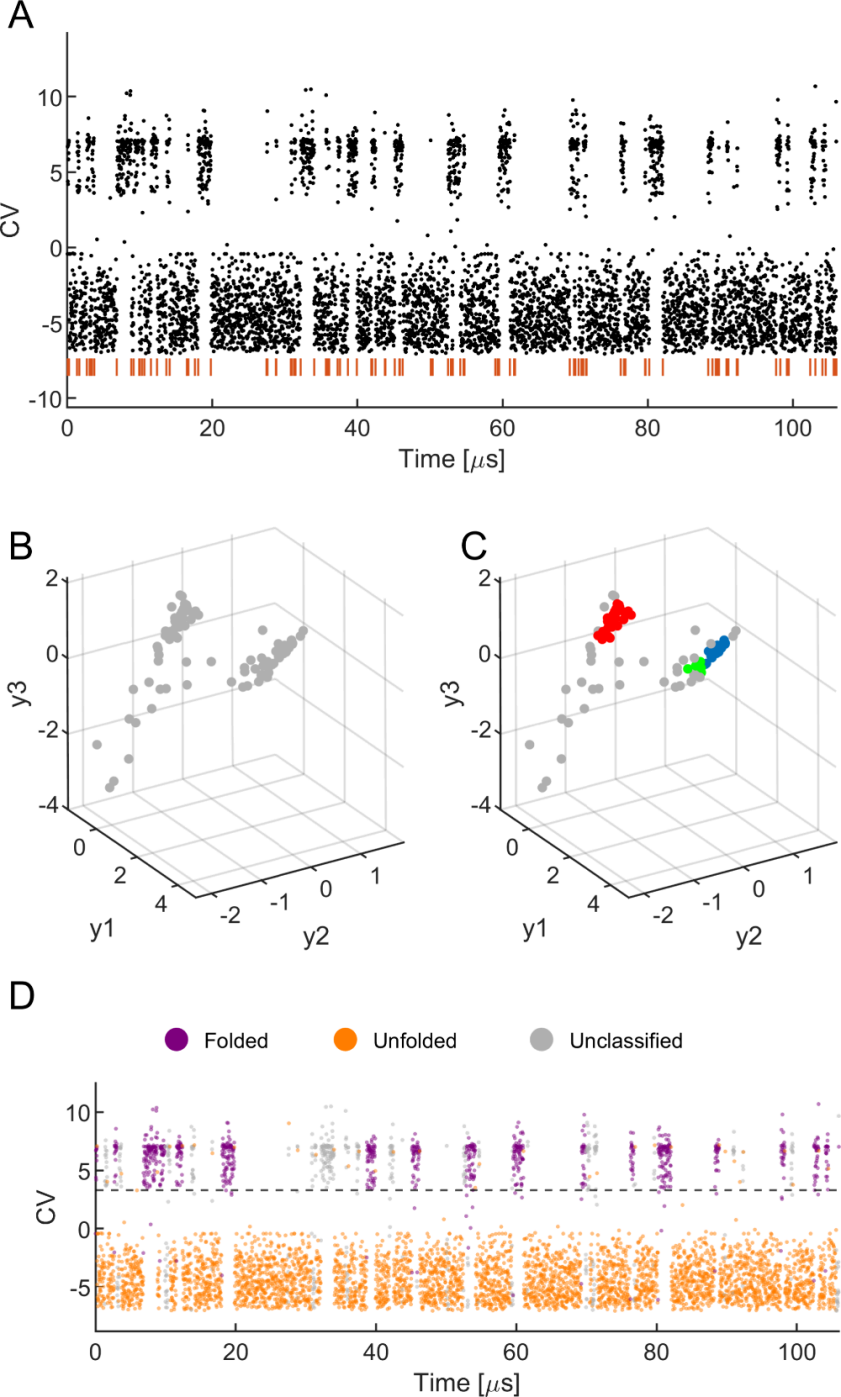
Protein state classification for CV _CH_^TPI-Deep-TDA^ of Chignolin data. (A) The CV _CH_^TPI-Deep-TDA^ value as a function of time (black dots), plotted every 20 ns, and the division into segments according to the trend change points detected by the BEAST algorithm (orange vertical lines). (B) Scatter plot for the Stochastic Landscape for the Chignolin trajectory, where each data point represents a segment corresponding to its stochastic coordinates *y*_1_, *y*_2_, and *y*_3_, which are the PCA components of the normalized mean, standard deviation, and average trend. (C) The result of the DBSCAN clustering algorithm depicted on the same scatter plot. Points in red, green, and blue correspond to different segment clusters, respectively, whereas unclassified segments remain in gray. (D) Protein state labeling, determined by the Kuhn-Munkres algorithm applied to the DBSCAN clustering results, projected onto the original CV _CH_^TPI-Deep-TDA^ trajectory. Purple and orange data points correspond to folded and unfolded states, respectively, whereas gray corresponds to unclassified samples. The horizontal dashed line is the ground-truth division between the folded and unfolded states.

The trajectory is initially downsampled by a factor of 10 to enhance computational efficiency. Next, the BEAST algorithm is applied to the downsampled trajectory, resulting in segmented data illustrated in Figure 1A. For each segment, stochastic coordinates are extracted, normalized, and subjected to principal component analysis (PCA) for dimensionality reduction. Utilizing these processed coordinates, we construct the Stochastic Landscape, depicted in Figure 1B, where each point in the scatter plot represents a segment as a function of the corresponding principal components of its stochastic coordinates. The DBSCAN algorithm then classifies the data based on heuristic inputs (detailed in section S2.1 of the SI), with the clustering outcome shown in Figure 1C. In this figure, classified data points belonging to different clusters are represented by different colors, whereas unclassified segments appear in gray. It is apparent that the segments classified as originating from the same state by the DBSCAN algorithm appear closer in the scatter plot, while unclassified data points appear to be more sporadically scattered. Employing the Kuhn-Munkres algorithm (as detailed in section 2.2 of the SI), clusters are labeled according to their corresponding ground-truth protein states. This step addresses the discrepancy between the number of identified clusters and the number of ground truth states, as the former is larger than the latter, as observed in Figure 1C. Finally, cluster labels are used for labeling the corresponding data points along the original trajectory. The outcome, including a threshold line indicating ground truth state classification, is presented in Figure 1D.

Visual comparison of the labeled states against the ground truth—illustrated by the dashed threshold line—qualitatively evaluates the classification performance. Specifically, data samples below the threshold line represent State 1, while those above indicate State 2. This comparison reveals high accuracy for State 2 classifications, with slightly lower performance for State 1, demonstrating the overall efficacy of our approach.

The results of the protein state classification applied to CV _CH_^Dist^, CV _CH_^RMSD^, CV _CH_^HLDA^, and CV _CH_^Deep-TDA^ are depicted in Figures S1, S2, S3, S4 in the SI.

**Figure 2:**
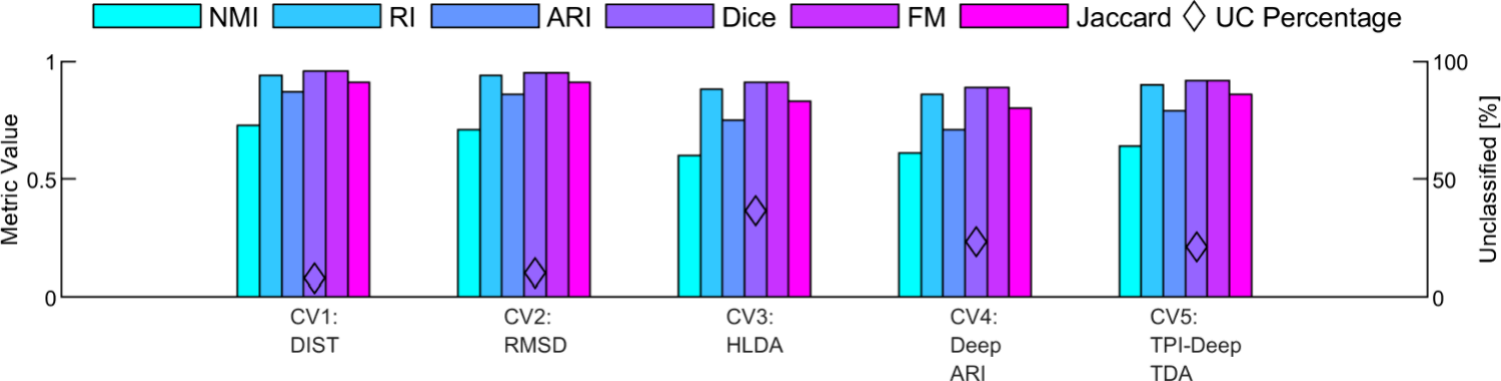
SLC results analysis for the Chignolin. The values of each of the 6 classification metrics are plotted for the 5 CVs of the Chignolin protein (left *y*-axis). The percentages of unclassified data (out of the 5348 samples in total) in each of the 5 CVs are shown as black diamonds (right *y*-axis).

To quantitatively assess the classification performance, we have computed the metrics previously discussed and presented the results in Figure 2. Most of these indices approach a value of 1, signifying a high level of classification accuracy. Notably, the NMI index exhibits a value significantly lower than the others. However, considering its nonlinear behavior, the NMI still reflects satisfactory performance. When comparing the classification metrics across different CVs of this trajectory, also detailed in Figure 2, it is evident that the results consistently indicate robust classification.^74^ Specifically, for all metrics other than the NMI, values are uniformly high, with none dropping below 0.71. Remarkably, CV1 demonstrates the highest classification accuracy across all evaluated metrics. This finding is particularly interesting, considering that the ground truth was initially established based on CV _CH_^TPI-Deep-TDA^, which is known to contain more information about the protein’s current state.^46^

Upon reviewing the classified trajectories of Chignolin data (as depicted in Figure 1D and Figures S1D, S2D, S3D, and S4D in the SI) alongside Figure 2, a clear correlation emerges between the evaluation metric values and the percentage of unclassified data segments (Figure S13 in the SI). Specifically, the correlation coefficients between the metric value and the unclassified data percentage are −0.93, – 0.81, – 0.81, – 0.78, – 0.78, and −0.8 for the NMI, RI, ARI, Dice, FM, and Jaccard metrics, respectively, suggesting that unclassified data negatively affects the results of the classification metrics, as expected. We hypothesize that the difference in unclassified data percentage of the CVs stems from the variability in the CV value statistics when dwelling in the same protein state on different occasions along the trajectory. This could result in different stochastic coordinates of segments belonging to the same protein state, hampering the classification task.

Addressing this classification challenge may require refining the DBSCAN algorithm’s heuristic parameters (as discussed in section S2.1 of the SI) to decrease the number of unclassified samples. Another approach could involve increasing the number of state visitations within the trajectory. The rationale is that a higher number of state visitations enriches the data set with more representative points for each state, potentially enhancing classification accuracy. This principle aligns with the SLC prerequisite for multiple state visitations (see Section S2.5 in the SI for minimal state visitation count analysis), underscoring the importance of capturing a wide range of statistical features to accurately characterize each state.

### 3.3 Metastable State Identification for Trp-Cage

#### 3.3.1 Ground Truth Data

Since the 3-state Deep-TDA CV (CV _TRP_^Deep-TDA-II^) has been trained explicitly to distinguish between the three metastable states of Trp-cage (folded, unfolded, and misfolded), projecting the MD trajectory in this space clearly distinguishes the configurations coming from these three states. CV _TRP_^Deep-TDA-II^ has been employed to define the ground truth for state classification in the following way. State 1, which corresponds to the folded state, is defined by CV _TRP_^Deep-TDA-II^ values smaller than −4. State 2, which corresponds to the unfolded state, includes CV _TRP_^Deep-TDA-II^ values in the range of [−2, 3]. State 3, which corresponds to the misfolded state, is defined by CV _TRP_^Deep-TDA-II^ values greater than 5. The categorization of samples with CV values that fall within the intermediate ranges, [−4, – 2] and [3,5] remains indeterminate. The boundaries of the different states in the Deep-TDA CV space are chosen to include all the training and test data points belonging to the corresponding states (see details of the Deep-TDA training protocol in ref (45).). For the purpose of establishing a clear basis for comparison, the classification is arbitrarily set such that samples with a CV _TRP_^Deep-TDA-II^ value below −2 are allocated to State 1 (folded), those within the range [−2, 3] are classified as State 2 (unfolded), and samples with a CV _TRP_^Deep-TDA-II^ value above 3 are assigned to State 3 (misfolded).

This allows for the systematic labeling of trajectory samples based on their respective states. The sequence of these sample labels as a function of time forms the ground truth for evaluating the classification results for all considered CVs.

#### 3.3.2 The SLC Implementation

**Figure 3:**
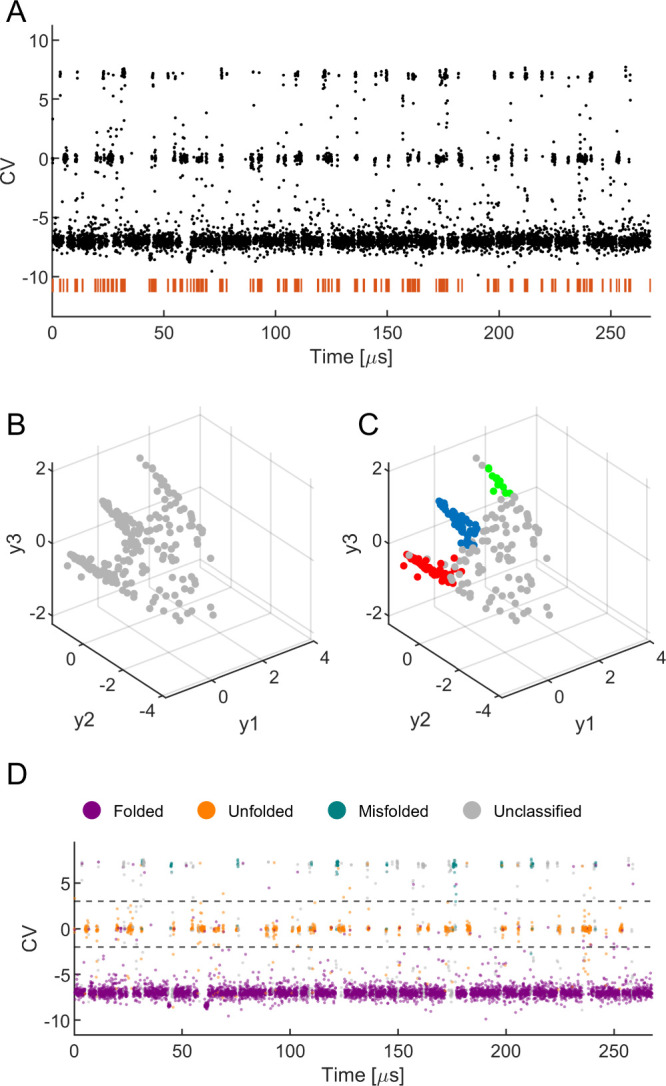
Protein state classification for CV _TRP_^Deep-TDA-II^ of Trp-Cage data. (A) The CV _TRP_^Deep-TDA-II^ value as a function of time (black dots), plotted every 40 ns, and its division into segments by the trend change points detected by the BEAST algorithm (orange vertical lines). (B) Scatter plot for the Stochastic Landscape for the Trp-Cage trajectory, where each data point represents a segment corresponding to its stochastic coordinates *y*_1_, *y*_2_, and *y*_3_, which are the PCA components of the normalized mean, standard deviation, and average trend. (C) The result of the DBSCAN clustering algorithm depicted on the same scatter plot. Points in red, blue, and green correspond to different segment clusters, respectively, whereas unclassified segments remain in gray. (D) Protein state labeling, determined by the Kuhn-Munkres algorithm applied to the DBSCAN clustering results, projected onto the original CV _TRP_^Deep-TDA-II^ trajectory. Purple, orange, and teal data points correspond to folded, unfolded, and misfolded states, respectively, whereas gray corresponds to unclassified samples. The horizontal dashed lines are the ground-truth division between the folded, unfolded, and misfolded states.

The Stochastic Landscape Classification approach is applied to the Trp-Cage CVs, as outlined in Section 2.1.2. We begin with the implementation of the methodological framework on CV _TRP_^Deep-TDA-II^ trajectory, chosen as the input. The trajectory undergoes downsampling by a factor of 8, reducing the data volume for more efficient processing without significantly compromising the informational content of the trajectory. Next, the BEAST algorithm is applied to the downsampled data, resulting in a segmented representation of the trajectory, as shown in Figure 3A.

The stochastic coordinates of each segment are then extracted, normalized, and subjected to principal component analysis (PCA) to distill the key features and reduce the dimensionality of the data. With the transformed data, the Stochastic Landscape is constructed, as showcased in Figure 3B. Utilizing the DBSCAN algorithm and heuristic inputs (detailed in Section S2.1 of the SI), the data is classified into clusters, reflecting the underlying data structure as depicted in Figure 3C. The clusters are then labeled according to their corresponding protein states based on a comparison with ground truth data, following the Kuhn-Munkres algorithm (see Section 2.2 of the SI). Finally, the clusters are mapped back to their original data points along the input trajectory. They are colored based on their labels and visualized with the threshold lines of the ground truth values, as illustrated in Figure 3D.

A visual examination of the state labels, represented by different colors against the dashed threshold lines in Figure 3D, provides a qualitative assessment of the classification performance. The dashed lines serve as demarcation points for the ground truth states, where samples found below the lower dashed line correspond to State 1 (folded), samples between the dashed lines correspond to State 2 (unfolded), and samples above the upper dashed line correspond to State 3 (misfolded). This visual comparison reveals that the classification performance for states 1 and 2 is notably effective, with the majority of samples accurately labeled in accordance with their respective states. However, while there are instances where samples belonging to state 3 are correctly classified, it is also observed that some of these samples are either mislabeled or remain unlabeled.

**Figure 4:**
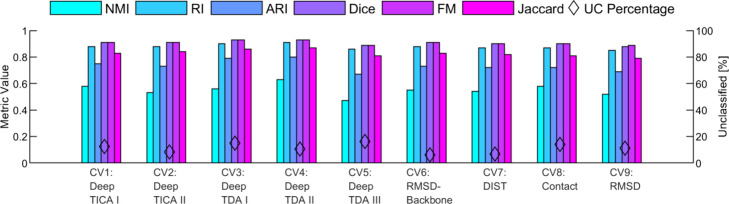
SLC results analysis for the Trp-Cage. The values of each of the 6 classification metrics are plotted for the 9 CVs of the Trp-Cage protein (left *y*-axis). The percentages of unclassified data (out of the 6692 samples in total) in each of the 9 CVs are shown as black diamonds (right *y*-axis).

To quantitatively assess the classification performance compared to the ground truth, the evaluation metrics are computed and presented in Figure 4. Most indices approach the value of 1, indicating a robust classification performance. While the NMI metric is lower than other indices, it still performs well. The highest classification accuracy is for CV _TRP_^Deep-TDA-II^, underscoring its distinct effectiveness in capturing relevant features for classification purposes. Nonetheless, the other CVs also demonstrate notable classification capabilities in comparison to the ground truth.

Unlike the Chignolin, where variation in classification performance could be attributed to the different percentage of unclassified data points, all CVs in the case of Trp-Cage show a similar amount of unclassified data, suggesting that the variances in classification success rates might be related to the inherent informational content of each CV. For a detailed examination of the classification performance of other CVs of Trp-Cage see Figures S5–S12 in the SI.

**Figure 5:**
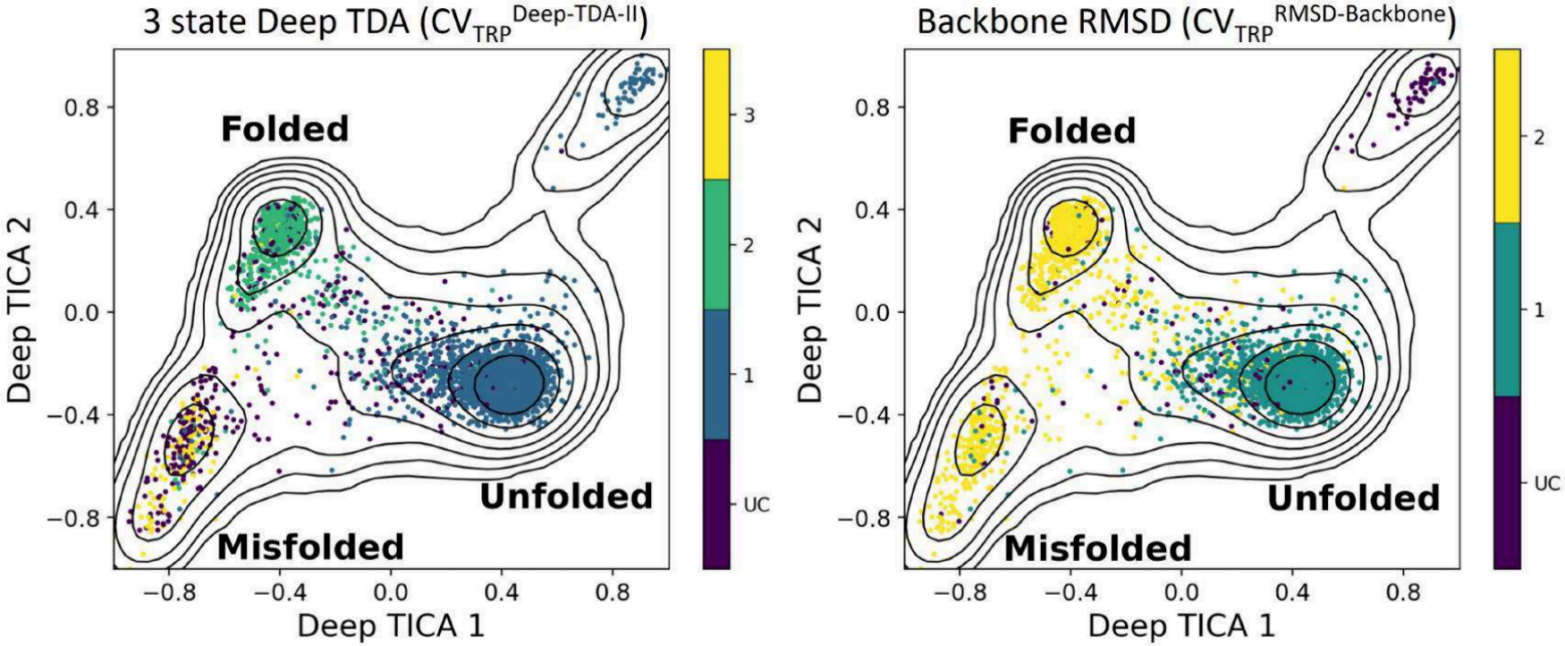
Projection of the molecular configurations clustered using of CV _TRP_^Deep-TDA-II^ (left) and CV _TRP_^RMSD-Backbone^ (right) on the free energy landscape of Trp-cage protein. The black lines represent the contour plot of the free energy surface obtained from the unbiased MD simulation in ref (62). The free energy surface is plotted as a function of the two slowest degrees of freedom (CV1 and CV2) obtained from deep-TICA analysis. The numbers 1, 2, and 3 indicate cluster indices, and UC indicates data points that could not be classified. In the left plot, SLC clustering based on Deep TDA CV could correctly classify the data points into folded, unfolded, and misfolded configurations. However, the same based on RMSD could not distinguish between the folded and misfolded states.

The free-energy landscape of the Trp-Cage protein obtained from unbiased MD simulation of Trp-Cage by Piana et al.^62^ is shown in Figure 5. This map is based on two principal collective variables, CV _TRP_^Deep-TICA-I^ and CV _TRP_^Deep-TICA-II^, and is categorized by clustered data points generated by the SLC of the advanced CV _TRP_^Deep-TDA-II^ (Deep-TDA) as well as the traditional (backbone RMSD). Despite having a comparable classification accuracy with more advanced CVs (CV _TRP_^Deep-TDA-II^), the backbone RMSD CV (CV _TRP_^RMSD-Backbone^) could not identify the three different free energy minima in the conformational landscape of Trp-Cage. Therefore, the identification of the three states using the SLC approach is more profound for CV _TRP_^Deep-TDA-II^ than CV _TRP_^RMSD-Backbone^. This aligns with previous findings^44^ that the deep-CVs (e.g., CV _TRP_^Deep-TDA-I^, CV _TRP_^Deep-TDA-II^, and CV _TRP_^Deep-TDA-III^ of Trp-Cage) are significantly better than the conventional CVs (e.g., CV _TRP_^RMSD-Backbone^, CV _TRP_^Dist^, CV _TRP_^Contact^, and CV _TRP_^RMSD^ of Trp-Cage) in accelerating transitions between the folded and unfolded states using enhanced sampling simulations. Notwithstanding, the high accuracy of the stochastic landscape classification for the conventional CVs indicates that it can be beneficial without the necessity of training a sophisticated neural network-based CV. Therefore, a refinement of the collective variables would contribute to enhanced results within the SLC method. Albeit the possibility to distinguish between only two of the protein states, the high classification metrics performance of the backbone RMSD CV compared to the 3-state Deep-TDA is attributed to the small number of visits in state 3 along the trajectory and their short dwelling times in these visitations (see Figure 3D). Similar to the Chignolin, it is possible that better identification of state 3 is achievable by the DBSCAN parameters variation.

## 4 Conclusions

Our study introduces a novel unsupervised methodology for assigning protein states along a dynamic trajectory realized by molecular dynamics (MD) simulations. This approach conceptualizes state classification within protein structures as a clustering problem, and evaluates its effectiveness with established classification metrics. By investigating the trajectories of Chignolin and Trp-Cage proteins, we demonstrate that accurate state classification can be achieved without the need for learning algorithms, provided there are frequent visits to these states within a trajectory and the statistical features of the states remain constant over time. This approach leverages the inherent statistical properties of protein states and their dynamic transitions. The first step identifies state-switching events by analyzing the trend changes in the CV trajectories. These trend changes act as dynamic order parameters, effectively segmenting the CV data into distinct epochs corresponding to different protein states. The second step exploits the time-invariant nature of protein state statistics. Since a protein occupies the same state with similar statistical properties upon multiple visits, we apply a clustering algorithm to the segmented CV data in the stochastic coordinates space. This allows us to assign each segment to a specific protein state based on the similarity of their statistical features. The significant accuracy presented for the two proteins demonstrates the potential of our approach as a generalized tool for analyzing protein dynamics and identifying conformational states. Overall, this methodology holds the potential for analyzing trajectories originating from experimental data and for various Collective Variables (CVs) that only contain partial information about the system.

From a computational perspective, we propose strategies for managing the analysis of long trajectories, including downsampling or employing our classification approach on a segment-by-segment basis. Central to our approach is the BEAST algorithm, which utilizes one-dimensional time-series data for effective classification. This algorithm is noteworthy for its ability to match the accuracy of structure-based clustering algorithms while integrating kinetic information into the clustering process. This feature is crucial for understanding the kinetic stability of biomolecules, making the BEAST algorithm a versatile tool for analyzing a variety of MD trajectory realization of biomolecules dynamics. Furthermore, the BEAST algorithm only acts on the time series data, making it independent of the relative probabilities of the configurations visited, as long as the minimal visitation count per state prerequisite is achieved (Section S2.5 in the SI). Therefore, our algorithm is indifferent to the underlying Hamiltonian of the dynamics and can be trivially extended to biased trajectories obtained from enhanced sampling simulations. Modern adaptive bias-enhanced sampling methods like On-the-fly Probability Enhanced Sampling (OPES)^81^ can quickly converge the external bias potential. MD trajectories obtained from OPES simulation are, therefore, in equilibrium with the rare events being accelerated. The SLC can thus directly analyze these trajectories to cluster the sampled metastable configurations.

The SLC can classify the states of a set of short trajectories as well by concatenating them together into a single long trajectory and then performing the classification. This approach is valid under two conditions. First, the concatenation process should be conducted carefully with truncation of each trajectory in the exact time step of a state change. This would ensure that the sampled state-dwelling times indeed follow their real statistics. If the latter cannot be performed, this would possibly result in a physically unlikely long trajectory. This undesired effect can be further mitigated if the trajectories state visitation count increases. Second, the underlying folding dynamics should be ergodic. Future work might shed light on how the SLC can be extended to classify states of short trajectories with nonergodic dynamics.

Looking ahead, we aim to extend the application of our methodology to more complex proteins exhibiting multiple states, such as the α3*D*^82^ and others, and to validate its effectiveness in experimental contexts, such as the study of intrinsically disordered proteins (IDPs).^83^ Given that the associated CVs contain relevant dynamic information, we expect that the SLC would perform well for such multiple-state proteins. Nevertheless, the SLC performance dependence on the CV trajectory length needs to be considered (See Section S2.5 in the SI). We also plan to explore other unsupervised classification algorithms to enhance the SLC’s performance, especially in scenarios involving nonstationary protein state statistics, like those induced by environmental changes such as temperature fluctuations. The SLC is designed to be a comprehensive automated tool for identifying protein states in both computational and experimental settings, offering a new perspective to exploring protein folding dynamics. It enables the evaluation of various CVs across different proteins and trajectories, thereby enhancing our understanding of protein dynamics. Our methodology opens new avenues for applying protein folding dynamics in industrial contexts, such as engineering more stable enzymes for cost-effective drug synthesis^84^ and developing enzymes for the efficient processing of plant biomass.^85,86^

In summary, our work underscores the significant potential of SLC in the realm of protein folding analysis, offering a novel and effective method for classifying protein states, understanding the informational content of CVs, and tracking statistical variations over time. This study not only deepens our understanding of protein dynamics but also sets the stage for future research in this field.

## Data Availability

The data underlying this study and open source codes are openly available in the GitHub repository of the paper.^63^

## Supporting Information Available

The BEAST algorithm details, additional details of the SLC implementation on protein folding, Figure S1: The BEAST algorithm activation on CV _CH_^Dist^ of the Chignolin data, Figure S2: The BEAST algorithm activation on CV _CH_^RMSD^ of the Chignolin data, Figure S3: The BEAST algorithm activation on CV _CH_^HLDA^ of the Chignolin data, Figure S4: The BEAST algorithm activation on CV _CH_^Deep-TDA^ of the Chignolin data, Figure S5: The BEAST algorithm activation on CV _TRP_^Deep-TICA-I^ of the Trp-Cage data, Figure S6: The BEAST algorithm activation on CV _TRP_^Deep-TICA-II^ of the Trp-Cage data, Figure S7: The BEAST algorithm activation on CV _TRP_^Deep-TDA-I^ of the Trp-Cage data, Figure S8: The BEAST algorithm activation on CV _TRP_^Deep-TDA-III^ of the Trp-Cage data, Figure S9: The BEAST algorithm activation on CV _TRP_^RMSD-Backbone^ of the Trp-Cage data, Figure S10: The BEAST algorithm activation on CV _TRP_^Dist^ of the Trp-Cage data, Figure S11: The BEAST algorithm activation on CV _TRP_^Contact^ of the Trp-Cage data, Figure S12: The BEAST algorithm activation on CV _TRP_^RMSD^ of the Trp-Cage data, Figure S13: The metric values of the SLC performance for the Chignolin classification as a function of the unclassified data percentage for CV1, CV2, CV3, CV4, and CV5. Figure S14: Classification metric values and state visitations versus the CV trajectory length (PDF)
