# Correction to A Stochastic Landscape Approach for Protein Folding State Classification

**DOI:** 10.1021/acs.jctc.4c00876

**Published:** 2024-08-12

**Authors:** Michael Faran, Dhiman Ray, Shubhadeep Nag, Umberto Raucci, Michele Parrinello, Gili Bisker

The correct reference to the Stochastic Landscape Classification (SLC) open-source software (ref 63) is as follows:

FaranM.A Stochastic Landscape Approach for Protein Folding State Classification. 2024. https://github.com/michaelfaran/A-Stochastic-Landscape-Approach-for-Protein-Folding-State-Classification (Accessed 2024-06-24).10.1021/acs.jctc.4c00464PMC1123853838924770

