# Supplementary material for: A Stochastic Landscape Approach for Protein Folding State Classification
Source: J Chem Theory Comput. 2024 Jun 26;20(13):5428–38. doi: 10.1021/acs.jctc.4c00464 (PMC11238538; doi:10.1021/acs.jctc.4c00464)
Supplement: Supplementary file 1 — ct4c00464_si_001.pdf [file ct4c00464_si_001.pdf]

Supporting Information: A Stochastic Landscape Approach for Protein Folding State Classification

Michael Faran,† Dhiman Ray,‡ Shubhadeep Nag,† Umberto Raucci,‡ Michelle Parrinello,‡ and Gili Bisker*,†,¶,§,‖

†*Department of Biomedical Engineering, Faculty of Engineering, Tel Aviv University, Tel Aviv 69978, Israel*

‡*Atomistic Simulations, Italian Institute of Technology, Via Enrico Melen 83, 16152 Genova, Italy*

¶*The Center for Physics and Chemistry of Living Systems, Tel Aviv University, Tel Aviv 6997801, Israel*

§*The Center for Nanoscience and Nanotechnology, Tel Aviv University, Tel Aviv 6997801, Israel*

‖*The Center for Light-Matter Interaction, Tel Aviv University, Tel Aviv 6997801, Israel*

E-mail: bisker@tauex.tau.ac.il

S1 The BEAST Algorithm Details

The BEAST algorithm deals with time series data $y_i \in \vec{y}$ with samples $i = 1 \ldots N_s$ where each sample is assumed to belong to one of several intervals $m_s = 1, 2 \ldots M_s$ with varying durations τ_{s,m_s}. These variables are represented altogether by a model vector $\vec{M}$. Intervals are further characterized by their trend and seasonality,[1] represented by a vector $\vec{\beta}_m$. A Gaussian noise $\epsilon = N(0, \sigma^2)$ is assumed for each interval, with a changing variance for each segment. The algorithm aims to find the optimal segmentation of the data by estimating the interval durations, trend and seasonality parameters, and noise variance. This is achieved through Bayesian inference, which involves maximizing the posterior probability of the model parameters given the observed data, $P_{pr} = p(\vec{M}, \vec{\beta}_m, \sigma^2|\vec{y})$. The posterior probability is calculated by combining the likelihood probability (based on the assumption of Gaussian noise) with prior probabilities based on these assumptions:

- Uniform prior the number of change-points in $\vec{M}$ with a large upper bound.
- Uniform prior for the intervals duration in $\vec{M}$ with a minimum length constraint.
- Normal-inverse Gamma prior for the noise variance amplitude, σ^2.
- The trend and seasonality parameters that are normally distributed with the resulting variance of σ^2.

While the posterior probability has a closed-form expression, finding the optimal parameters analytically is impossible. Therefore, the BEAST algorithm utilizes a Reverse Jump Markov chain Monte Carlo (MCMC) sampler within a Gibbs sampling framework to estimate the parameters iteratively. This eventually leads to optimal estimates of interval duration, trend and seasonality parameters, and noise variance. For additional details, please refer to the work of Zhao *et. al.*[1]

S2 Implementation of the Stochastic Landscape Classification on Protein Folding

The Stochastic Landscape Classification (SLC) implementation follows the steps described in Table. 1 in the main text. Additional information about the DBCAN classification and the Khun-Munkres label matching is given below.

S2.1 DBSCAN classification

While the CV segments are scattered in space (see Figs. 1B and 3B) vs. the stochastic coordinates, we aim to cluster them according to their position. This follows the assumption that different macro-states have different stochastic coordinates, originating in their respective different mean, STD, and average linear trends. The DBSCAN (Density-based spatial clustering of applications with noise) algorithm[2,3] is used widely for numerous classification tasks, such as superpixel segmentation,[4] radar data clustering,[5] gene clustering,[6] anomaly detection[7] and others.[8] This algorithm analyzes data points in a given space and identifies clusters based on their density. Clustered points are assumed to be closely packed, with many surrounding neighbors. Points located in areas with low density, those further away from their nearest neighbors, are classified as outliers by the algorithm. All these outliers are clustered with the label UC (unclassified).

Two parameters control the behavior of DBSCAN: $minPts$, which defines the minimum number of neighbors needed for a dense cluster, and ϵ, which sets the search radius for finding neighbors. We chose these parameters based on commonly used DBSCAN heuristics.[9] While for most applications, the $minPts$ value is suggested as twice the space dimension d, we choose $minPts = 2 \cdot d - 1 = 5$, where $d = 3$ (the stochastic landscape dimensionality). The ϵ value was heuristically chosen according to the knee point in a k-distance graph[2]. The knee point was determined using the following algorithm. First, move iteratively along the k-distance curve, examining one bisection point at a time. At each point, fit two lines, one

to all points left of the current point and another to all points to the right. The "knee" is then identified as the bisection point where the sum of errors for these two line fits is minimized.[10] After this classification, the cluster labels are assigned to each of the points in the scatter plots (see Figs. 1C and 3C of the main text). One cluster accounts for all the points considered as outliers. Finally, the labels are matched according to the Khun-Munkres algorithm.

S2.2 Khun-Munkres Label Matching

After applying the DBSCAN algorithm to the stochastic landscape data, labels are assigned to each point in the scatter plot based on the suggested clustering scheme. Subsequently, a comparison with the ground-truth data is pursued, giving rise to two challenges. First, discrepancies in the number of clusters may arise between the ground truth and the clustered data. Second, establishing the correspondence between labels in the clustered scheme and those in the ground truth necessitates attention. Addressing a similar issue, Chen et al.[11] proposed employing the Hungarian algorithm[12](also known as the Kuhn-Munkres algorithm), aiming to match labels akin to solving an assignment problem. This approach facilitates a comparison of labels between clusters and states in the ground truth. Notably, if the number of states in the ground truth differs from the number of clusters identified by DBSCAN, the number of matched labels is constrained by the minimum of the two cluster counts. Consequently, the remaining clusters and their associated points are amalgamated with the outliers cluster (UC). Subsequently, the segment labels are generated and utilized as colors in scatter plots (refer to Figs. 1C and 1D for Chignolin protein, and Figs. 3C and 3D for Trp-Cage protein).

In the context of this study, the clustering method was evaluated against a known ground truth. However, in applications where the ground truth is unknown, the label-matching scheme is irrelevant, and clusters should be interpreted based on the output of the DBSCAN algorithm, as described earlier.

S2.3 Additional CVs Segmented Trajectories

Following the SLC outline in Table. 1 of the main text, the following segmented CVs are depicted below for both the Chignolin and the Trp-Cage. Figures depicting protein state classification of Chignolin protein ($\mathrm{CV}_{\mathrm{CH}}^{\mathrm{Dist}}$, $\mathrm{CV}_{\mathrm{CH}}^{\mathrm{RMSD}}$, $\mathrm{CV}_{\mathrm{CH}}^{\mathrm{HLDA}}$, and $\mathrm{CV}_{\mathrm{CH}}^{\mathrm{Deep\text{-}TDA}}$) can be seen in Figs. S1, S2, S3, and S4, respectively. Similarly, for Trp-Cage, the same is depicted for $\mathrm{CV}_{\mathrm{TRP}}^{\mathrm{Deep\text{-}TICA\text{-}I}}$, $\mathrm{CV}_{\mathrm{TRP}}^{\mathrm{Deep\text{-}TICA\text{-}II}}$, $\mathrm{CV}_{\mathrm{TRP}}^{\mathrm{Deep\text{-}TDA\text{-}I}}$, $\mathrm{CV}_{\mathrm{TRP}}^{\mathrm{Deep\text{-}TDA\text{-}III}}$, $\mathrm{CV}_{\mathrm{TRP}}^{\mathrm{RMSD\text{-}Backbone}}$, $\mathrm{CV}_{\mathrm{TRP}}^{\mathrm{Dist}}$, $\mathrm{CV}_{\mathrm{TRP}}^{\mathrm{Contact}}$, $\mathrm{CV}_{\mathrm{TRP}}^{\mathrm{RMSD}}$ in Figs. S5, S6, S7, S8, S9, S10, S11, and S12, respectively.

S2.4 Chignolin Metric Values vs. Unclassified Data

Fig. S13 shows the resulting correlation between the classification metric values and the unclassified data percentage. In all cases, the Pearson coefficients[13] indicate a negative correlation.

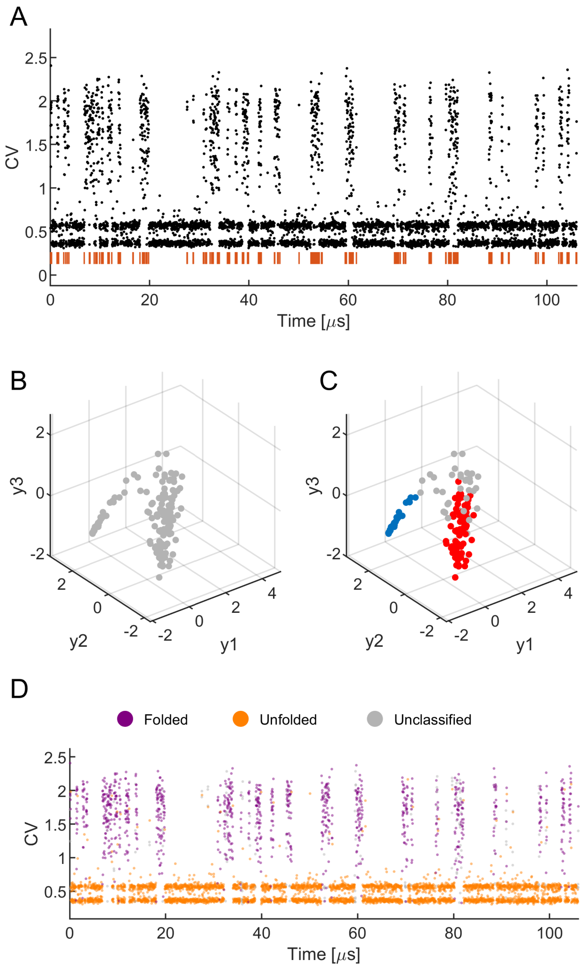

Figure S1: Protein state classification for $\mathrm{CV}_{\mathrm{CH}}^{\mathrm{Dist}}$ of Chignolin data. (A) The $\mathrm{CV}_{\mathrm{CH}}^{\mathrm{Dist}}$ value as a function of time (black dots), plotted every 20 ns, and the division into segments according to the trend change points detected by the BEAST algorithm (orange vertical lines). (B) Scatter plot for the Stochastic Landscape for the Chignolin trajectory, where each data point represents a segment corresponding to its stochastic coordinates y_1, y_2, and y_3, which are the PCA components of the normalized mean, standard deviation, and average trend. (C) The result of the DBSCAN clustering algorithm depicted on the same scatter plot. Points in red and blue correspond to different segment clusters, respectively, whereas unclassified segments remain in grey. (D) Protein state labeling, determined by the Kuhn-Munkres algorithm applied to the DBSCAN clustering results, projected onto the original $\mathrm{CV}_{\mathrm{CH}}^{\mathrm{Dist}}$ trajectory. Purple and orange data points correspond to folded and unfolded states, respectively, whereas grey corresponds to unclassified samples.

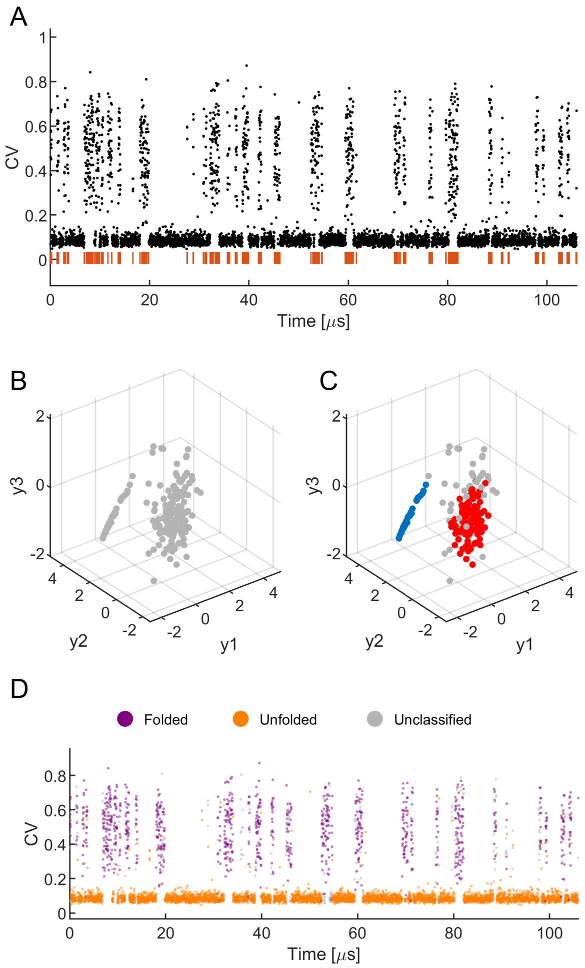

Figure S2: Protein state classification for CV_{CH}^{RMSD} of Chignolin data. (A) The CV_{CH}^{RMSD} value as a function of time (black dots), plotted every 20 ns, and the division into segments according to the trend change points detected by the BEAST algorithm (orange vertical lines). (B) Scatter plot for the Stochastic Landscape for the Chignolin trajectory, where each data point represents a segment corresponding to its stochastic coordinates y_1, y_2, and y_3, which are the PCA components of the normalized mean, standard deviation, and average trend. (C) The result of the DBSCAN clustering algorithm depicted on the same scatter plot. Points in red and blue correspond to different segment clusters, respectively, whereas unclassified segments remain in grey. (D) Protein state labeling, determined by the Kuhn-Munkres algorithm applied to the DBSCAN clustering results, projected onto the original CV_{CH}^{RMSD} trajectory. Purple and orange data points correspond to folded and unfolded states, respectively, whereas grey corresponds to unclassified samples.

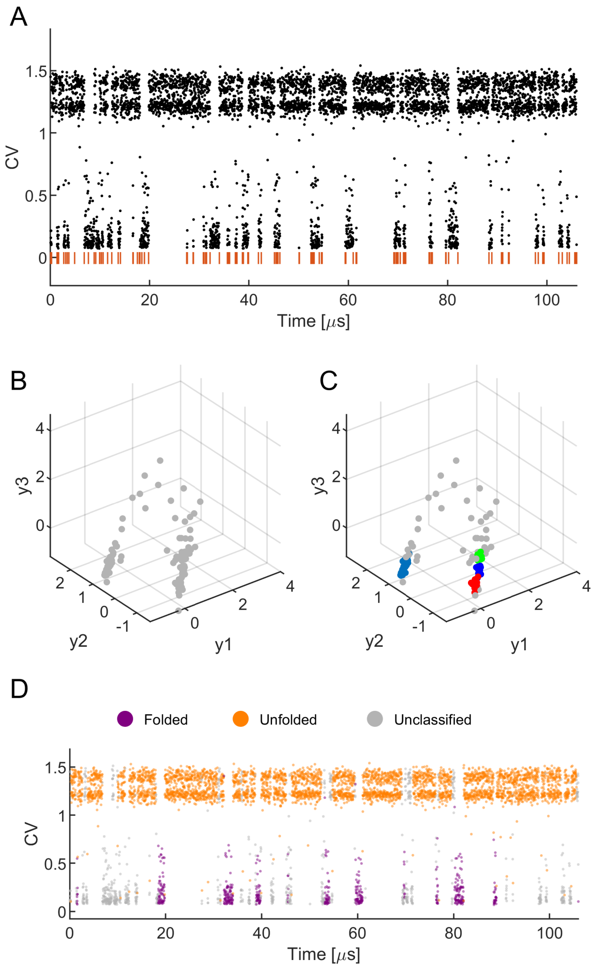

Figure S3: Protein state classification for CV_{CH}^{HLDA} of Chignolin data. (A) The CV_{CH}^{HLDA} value as a function of time (black dots), plotted every 20 ns, and the division into segments according to the trend change points detected by the BEAST algorithm (orange vertical lines). (B) Scatter plot for the Stochastic Landscape for the Chignolin trajectory, where each data point represents a segment corresponding to its stochastic coordinates y_1, y_2, and y_3, which are the PCA components of the normalized mean, standard deviation, and average trend. (C) The result of the DBSCAN clustering algorithm depicted on the same scatter plot. Points in red, light blue, green, and dark blue correspond to different segment clusters, respectively, whereas unclassified segments remain in grey. (D) Protein state labeling, determined by the Kuhn-Munkres algorithm applied to the DBSCAN clustering results, projected onto the original CV_{CH}^{HLDA} trajectory. Purple and orange data points correspond to folded and unfolded states, respectively, whereas grey corresponds to unclassified samples.

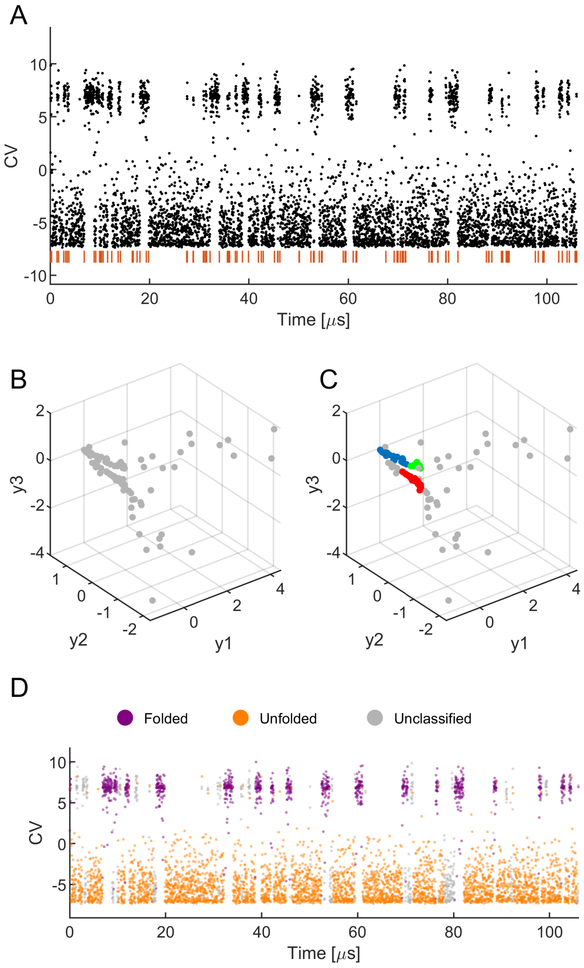

Figure S4: Protein state classification for $CV_{CH}^{TPI\text{-}Deep\text{-}TDA}$ of Chignolin data. (A) The $CV_{CH}^{TPI\text{-}Deep\text{-}TDA}$ value as a function of time (black dots), plotted every 20 ns, and the division into segments according to the trend change points detected by the BEAST algorithm (orange vertical lines). (B) Scatter plot for the Stochastic Landscape for the Chignolin trajectory, where each data point represents a segment corresponding to its stochastic coordinates y_1, y_2, and y_3, which are the PCA components of the normalized mean, standard deviation, and average trend. (C) The result of the DBSCAN clustering algorithm depicted on the same scatter plot. Points in red, blue, and green correspond to different segment clusters, respectively, whereas unclassified segments remain in grey. (D) Protein state labeling, determined by the Kuhn-Munkres algorithm applied to the DBSCAN clustering results, projected onto the original $CV_{CH}^{TPI\text{-}Deep\text{-}TDA}$ trajectory. Purple and orange data points correspond to folded and unfolded states, respectively, whereas grey corresponds to unclassified samples.

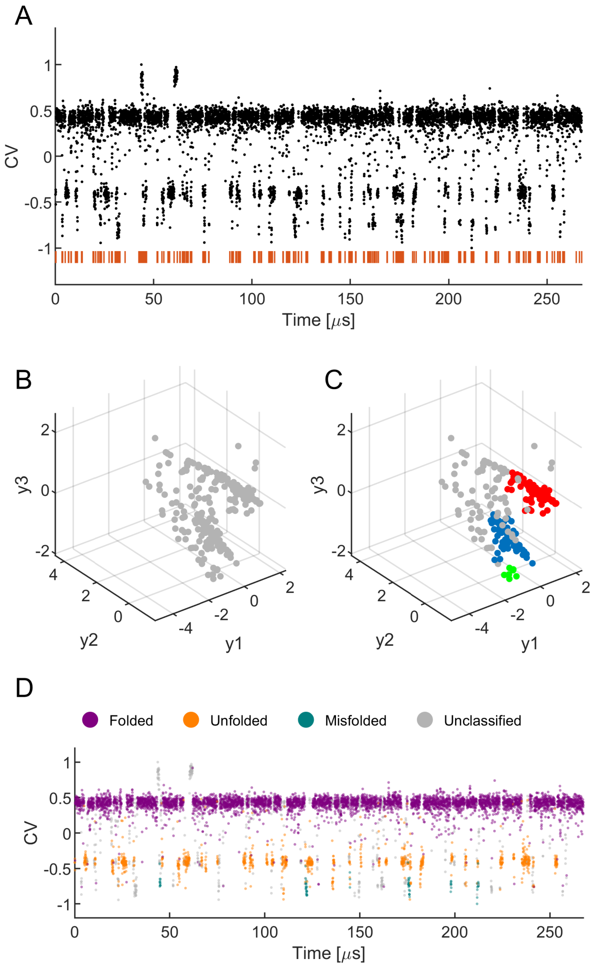

Figure S5: Protein state classification for $CV_{TRP}^{Deep\text{-}TICA\text{-}I}$ of Trp-Cage data. (A) The $CV_{TRP}^{Deep\text{-}TICA\text{-}I}$ value as a function of time (black dots), plotted every 40 ns, and the division into segments according to the trend change points detected by the BEAST algorithm (orange vertical lines). (B) Scatter plot for the Stochastic Landscape for the Trp-Cage trajectory, where each data point represents a segment corresponding to its stochastic coordinates y_1, y_2, and y_3, which are the PCA components of the normalized mean, standard deviation, and average trend. (C) The result of the DBSCAN clustering algorithm depicted on the same scatter plot. Points in red, blue, and green correspond to different segment clusters, respectively, whereas unclassified segments remain in grey. (D) Protein state labeling, determined by the Kuhn-Munkres algorithm applied to the DBSCAN clustering results, projected onto the original $CV_{TRP}^{Deep\text{-}TICA\text{-}I}$ trajectory. Purple, orange, and teal data points correspond to folded, unfolded, and misfolded states, respectively, whereas grey corresponds to unclassified samples.

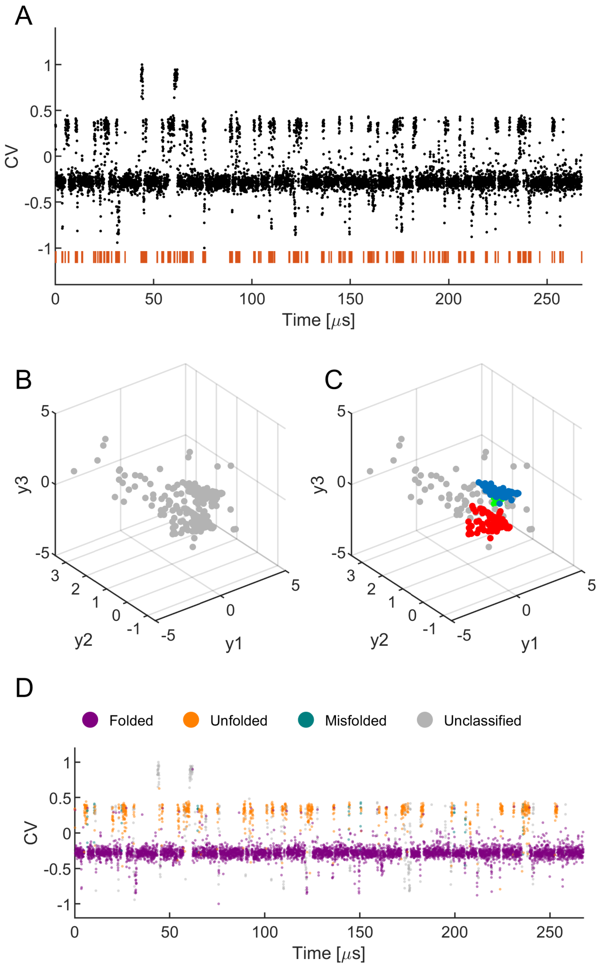

Figure S6: Protein state classification for $\mathrm{CV}_{\mathrm{TRP}}^{\text{Deep-TICA-II}}$ of Trp-Cage data. (A) The $\mathrm{CV}_{\mathrm{TRP}}^{\text{Deep-TICA-II}}$ value as a function of time (black dots), plotted every 40 ns,and the division into segments according to the trend change points detected by the BEAST algorithm (orange vertical lines). (B) Scatter plot for the Stochastic Landscape for the Trp-Cage trajectory, where each data point represents a segment corresponding to its stochastic coordinates y_1, y_2, and y_3, which are the PCA components of the normalized mean, standard deviation, and average trend. (C) The result of the DBSCAN clustering algorithm depicted on the same scatter plot. Points in red, blue, and green correspond to different segment clusters, respectively, whereas unclassified segments remain in grey. (D) Protein state labeling, determined by the Kuhn-Munkres algorithm applied to the DBSCAN clustering results, projected onto the original $\mathrm{CV}_{\mathrm{TRP}}^{\text{Deep-TICA-II}}$ trajectory. Purple, orange, and teal data points correspond to folded, unfolded, and misfolded states, respectively, whereas grey corresponds to unclassified samples.

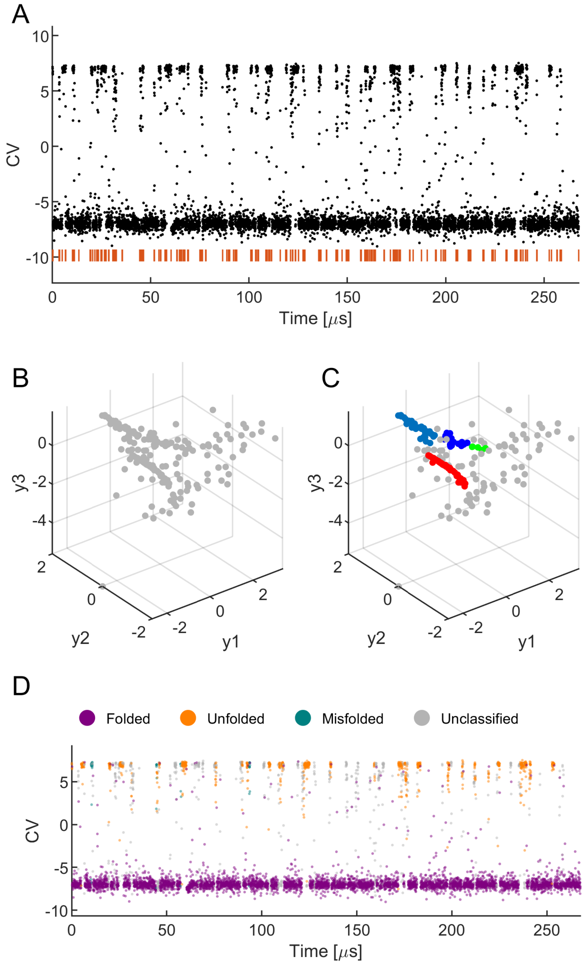

Figure S7: Protein state classification for $CV_{TRP}^{Deep\text{-}TDA\text{-}I}$ of Trp-Cage data. (A) The $CV_{TRP}^{Deep\text{-}TDA\text{-}I}$ value as a function of time (black dots), plotted every 40 ns, and the division into segments according to the trend change points detected by the BEAST algorithm (orange vertical lines). (B) Scatter plot for the Stochastic Landscape for the Trp-Cage trajectory, where each data point represents a segment corresponding to its stochastic coordinates y_1, y_2, and y_3, which are the PCA components of the normalized mean, standard deviation, and average trend. (C) The result of the DBSCAN clustering algorithm depicted on the same scatter plot. Points in red, light blue, dark blue, and green correspond to different segment clusters, respectively, whereas unclassified segments remain in grey. (D) Protein state labeling, determined by the Kuhn-Munkres algorithm applied to the DBSCAN clustering results, projected onto the original $CV_{TRP}^{Deep\text{-}TDA\text{-}I}$ trajectory. Purple, orange, and teal data points correspond to folded, unfolded, and misfolded states, respectively, whereas grey corresponds to unclassified samples.

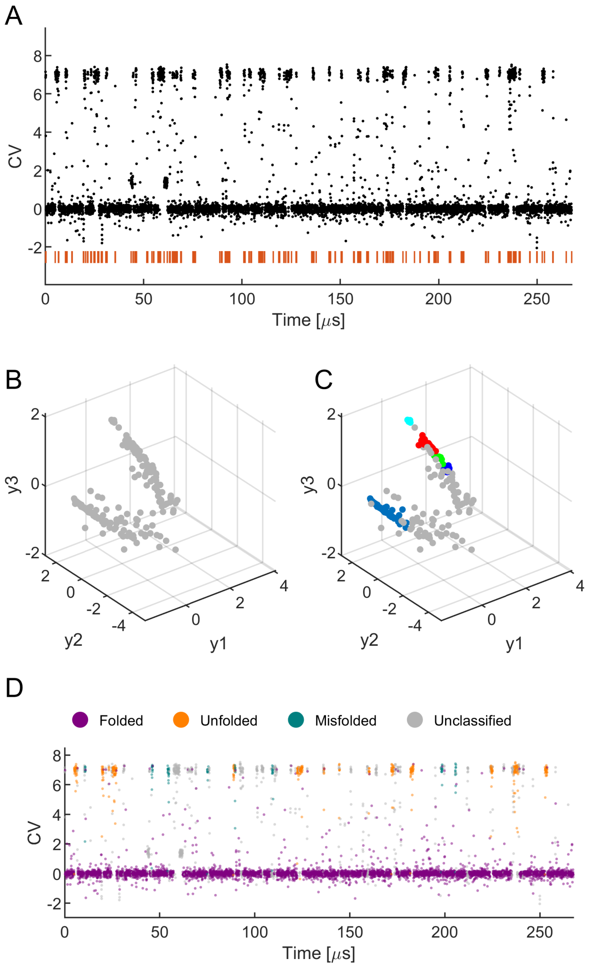

Figure S8: Protein state classification for $\mathrm{CV}_{\mathrm{TRP}}^{\text{Deep-TDA-III}}$ of Trp-Cage data. (A) The $\mathrm{CV}_{\mathrm{TRP}}^{\text{Deep-TDA-II}}$ value as a function of time (black dots), plotted every 40 ns, and the division into segments according to the trend change points detected by the BEAST algorithm (orange vertical lines). (B) Scatter plot for the Stochastic Landscape for the Trp-Cage trajectory, where each data point represents a segment corresponding to its stochastic coordinates y_1, y_2, and y_3, which are the PCA components of the normalized mean, standard deviation, and average trend. (C) The result of the DBSCAN clustering algorithm depicted on the same scatter plot. Points in red, dark blue, light blue, pale blue, and green correspond to different segment clusters, respectively, whereas unclassified segments remain in grey. (D) Protein state labeling, determined by the Kuhn-Munkres algorithm applied to the DBSCAN clustering results, projected onto the original $\mathrm{CV}_{\mathrm{TRP}}^{\text{Deep-TDA-II}}$ trajectory. Purple, orange, and teal data points correspond to folded, unfolded, and misfolded states, respectively, whereas grey corresponds to unclassified samples.

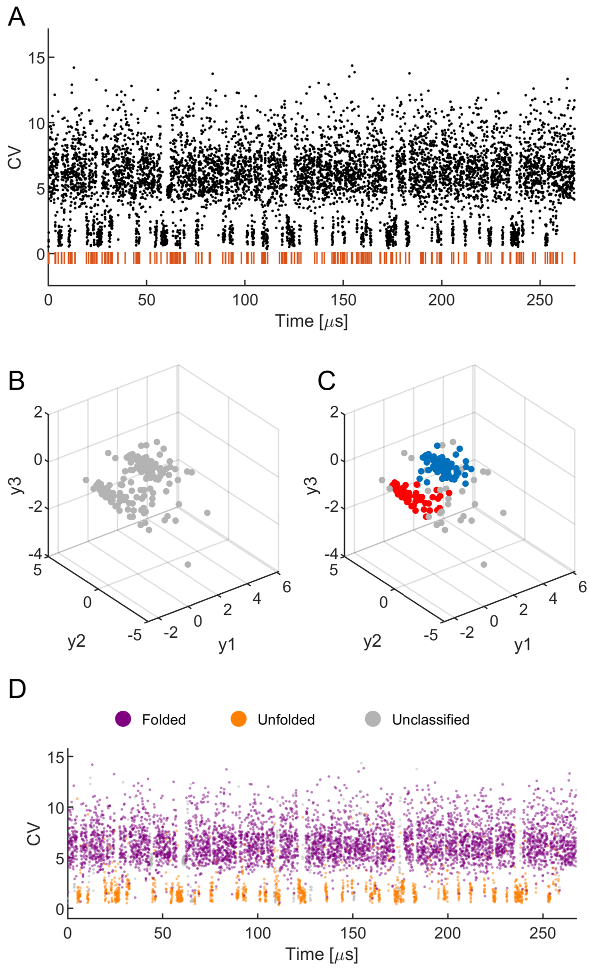

Figure S9: Protein state classification for $CV_{TRP}^{RMSD\text{-}Backbone}$ of Trp-Cage data. (A) The $CV_{TRP}^{RMSD\text{-}Backbone}$ value as a function of time (black dots), plotted every 40 ns, and the division into segments according to the trend change points detected by the BEAST algorithm (orange vertical lines). (B) Scatter plot for the Stochastic Landscape for the Trp-Cage trajectory, where each data point represents a segment corresponding to its stochastic coordinates y_1, y_2, and y_3, which are the PCA components of the normalized mean, standard deviation, and average trend. (C) The result of the DBSCAN clustering algorithm depicted on the same scatter plot. Points in red and blue correspond to different segment clusters, respectively, whereas unclassified segments remain in grey. (D) Protein state labeling, determined by the Kuhn-Munkres algorithm applied to the DBSCAN clustering results, projected onto the original $CV_{TRP}^{RMSD\text{-}Backbone}$ trajectory. Purple and orange data points correspond to folded and unfolded states, respectively, whereas grey corresponds to unclassified samples.

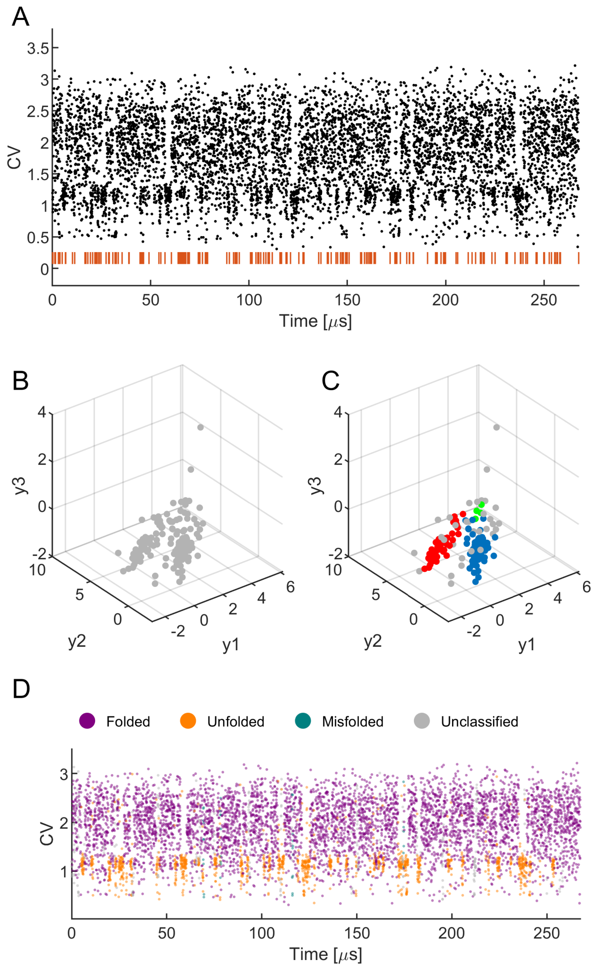

Figure S10: Protein state classification for CV_{TRP}^{Dist} of Trp-Cage data. (A) The CV_{TRP}^{Dist} value as a function of time (black dots), plotted every 40 ns, and the division into segments according to the trend change points detected by the BEAST algorithm (orange vertical lines). (B) Scatter plot for the Stochastic Landscape for the Trp-Cage trajectory, where each data point represents a segment corresponding to its stochastic coordinates y_1, y_2, and y_3, which are the PCA components of the normalized mean, standard deviation, and average trend. (C) The result of the DBSCAN clustering algorithm depicted on the same scatter plot. Points in red, blue, and green correspond to different segment clusters, respectively, whereas unclassified segments remain in grey. (D) Protein state labeling, determined by the Kuhn-Munkres algorithm applied to the DBSCAN clustering results, projected onto the original CV_{TRP}^{Dist} trajectory. Purple, orange, and teal data points correspond to folded, unfolded, and misfolded states, respectively, whereas grey corresponds to unclassified samples.

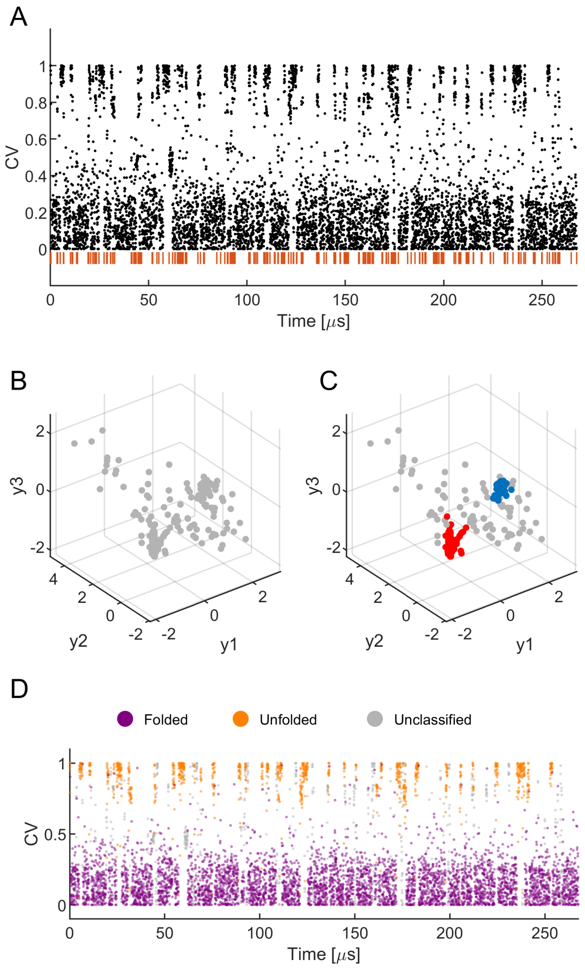

Figure S11: Protein state classification for $CV_{TRP}^{Contact}$ of Trp-Cage data. (A) The $CV_{TRP}^{Contact}$ value as a function of time (black dots), plotted every 40 ns, and the division into segments according to the trend change points detected by the BEAST algorithm (orange vertical lines). (B) Scatter plot for the Stochastic Landscape for the Trp-Cage trajectory, where each data point represents a segment corresponding to its stochastic coordinates y_1, y_2, and y_3, which are the PCA components of the normalized mean, standard deviation, and average trend. (C) The result of the DBSCAN clustering algorithm depicted on the same scatter plot. Points in red and blue correspond to different segment clusters, respectively, whereas unclassified segments remain in grey. (D) Protein state labeling, determined by the Kuhn-Munkres algorithm applied to the DBSCAN clustering results, projected onto the original $CV_{TRP}^{Contact}$ trajectory. Purple and orange data points correspond to folded and unfolded, respectively, whereas grey corresponds to unclassified samples.

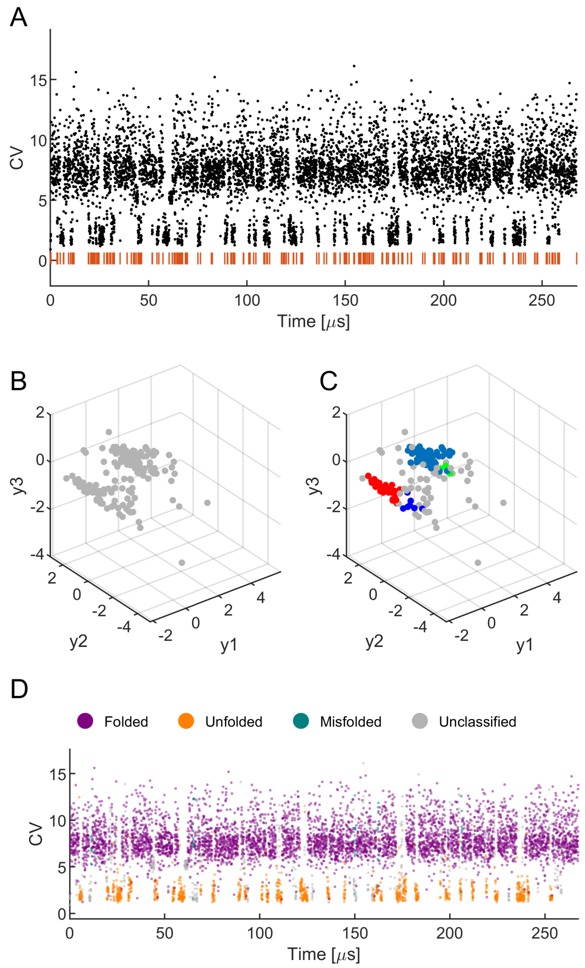

Figure S12: Protein state classification for CV_{TRP}^{RMSD} of Trp-Cage data. (A) The CV_{TRP}^{RMSD} value as a function of time (black dots), plotted every 40 ns, and the division into segments according to the trend change points detected by the BEAST algorithm (orange vertical lines). (B) Scatter plot for the Stochastic Landscape for the Trp-Cage trajectory, where each data point represents a segment corresponding to its stochastic coordinates y_1, y_2, and y_3, which are the PCA components of the normalized mean, standard deviation, and average trend. (C) The result of the DBSCAN clustering algorithm depicted on the same scatter plot. Points in red, light blue, dark blue, and green correspond to different segment clusters, respectively, whereas unclassified segments remain in grey. (D) Protein state labeling, determined by the Kuhn-Munkres algorithm applied to the DBSCAN clustering results, projected onto the original CV_{TRP}^{RMSD} trajectory. Purple, orange, and teal data points correspond to folded, unfolded, and misfolded states, respectively, whereas grey corresponds to unclassified samples.

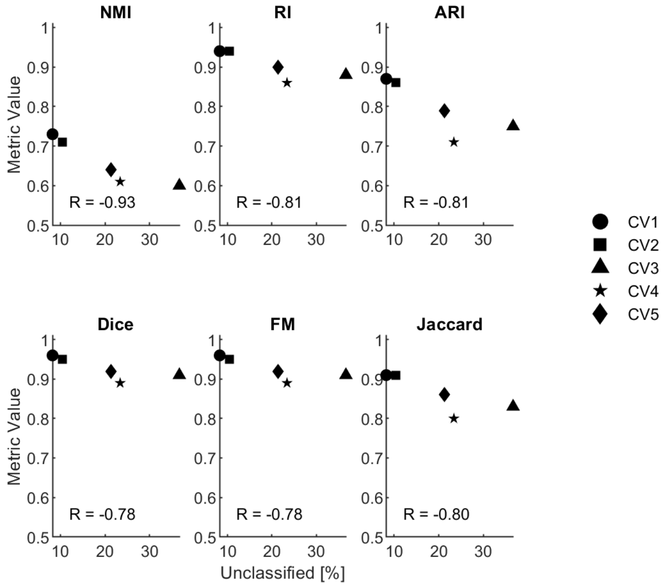

Figure S13: The metric values of the SLC performance for the Chignolin classification as a function of the unclassified data percentage for CV_{CH}^{Dist} (circles), CV_{CH}^{RMSD} (squares), CV_{CH}^{HLDA} (triangles), $CV_{CH}^{Deep\text{-}TDA}$ (stars), and $CV_{CH}^{TPI\text{-}Deep\text{-}TDA}$ (rhombuses). The Pearson correlation coefficients are listed for each of the metrics.

S2.5 Visitation Count and CV length Performance Analysis

In this work, the SLC was examined for CV trajectories containing numerous folded and unfolded state transitions. In the general sense, CV trajectories might contain none to a few state transitions. Aiming towards general applicability, we examine the minimal number of protein state visitations required for the SLC to perform well. Following the intuition of metastable basin transition causing trend change, each state visitation along the CV is characterized by at least one segment (occasionally, significant trend changes can occur while remaining in the metastable basin. For example, when a strong CV fluctuation is present, it might be insufficient to trigger an escape event). Therefore, each state visitation is associated with at least one scattered point in the stochastic landscape. The minimal number of points per cluster required by the DBSCAN algorithm is given by *minPts*. Since each classified cluster corresponds to a specific protein state and aims to capture all its associated scattered points within the stochastic landscape (see Section. S2.1), it is suggested that the

CV trajectory would contain at least *minPts* visitations for each state. Nevertheless, this lower bound suggestion is insufficient, since it assumes that no classification errors of the method exist when compared against the ground truth.

To attain the actual minimal number of state visitations required for the SLC to perform well, we have empirically tested the NMI and ARI classification metric values versus the state visitation counts (see Section 3.1 in the main text). For that cause, we chose $\mathrm{CV}_{\mathrm{CH}}^{\text{TPI-Deep-TDA}}$ of the Chignolin and $\mathrm{CV}_{\mathrm{TRP}}^{\text{Deep-TDA-II}}$ of the Trp-Cage. This comparison also examines the metric values versus the trajectory length, and appears in Fig. S14 A and B. The trajectory length is measured as the percentage of the original CV trajectory length for the two proteins. Sharp metric transitions occur at 9% and 4.5% trajectory lengths for the Chignolin (total length: 5348 samples) and the Trp-Cage (total length: 6692 samples) after downsampling (see Table 1 in the main text).

We propose that sharp metric transitions (Fig. S14 C and D) around the SLC minimum state visitation counts indicate the method's inability to classify states correctly before these transitions (near-zero metric values appear). For the two proteins, the SLC minimum state visitation count values are determined by the state visitation count plots intersection with the dashed vertical line in Fig. S14 C and D. For the Chignolin, the resulting minimal number of state visitation counts is 7 for both the folded and unfolded states. For the Trp-cage, the resulting minimal number of state visitation counts are 9, 8, and 3 visitations, respectively, for the folded, unfolded, and misfolded states. The latter number of visitations is less than *minPts* for the third state, which might seem counter-intuitive given the previous DBSCAN algorithm-based argument for the minimal visitation counts per state. This stems from the result of 9 and 8 visitations in the folded and unfolded states being sufficient to classify their clusters, regardless of the third state. This result yields, in this case, an already high value for the NMI and ARI classification metrics (0.62 and 0.79, respectively). This might be advantageous for some systems in which one protein state is rarely visited compared to the others. Here, for example, the third state can be regarded as merged with the unclassified

cluster (see section S 2.2). Together, they can be regarded as a complementary cluster to the two other classified protein states.

The required minimal number of state visitations can possibly be reduced by constructing the stochastic landscape as a $2D$ space instead of $3D$, using solely the first and second principal components as the standardized stochastic coordinates. This could occur since $minPts$ is calculated by $2 \cdot d - 1$, and hence, a smaller dimension would result in a reduced minimal data points requirement (or state visitations) per cluster. Nevertheless, this possibly comes with the cost of reduced classification accuracy, as some information would not be considered for the classification task. Above the yielded minimal number of state visitations, the classification metrics values versus the trajectories length show an unsteady performance (see Fig. S14 A and B), which is more pronounced for the Trp-Cage in Fig. S14 B, with dips appearing for certain values. Classification errors by the SLC (compared to ground truth) cause the observed bounded unsteadiness. Above the minimum state visitation counts, performance does not necessarily improve, suggesting either saturation or limitations in the SLC's data exploitation, which remains for future work examination. Albeit this bounded unsteadiness, the classification metric values are significantly greater than zero.

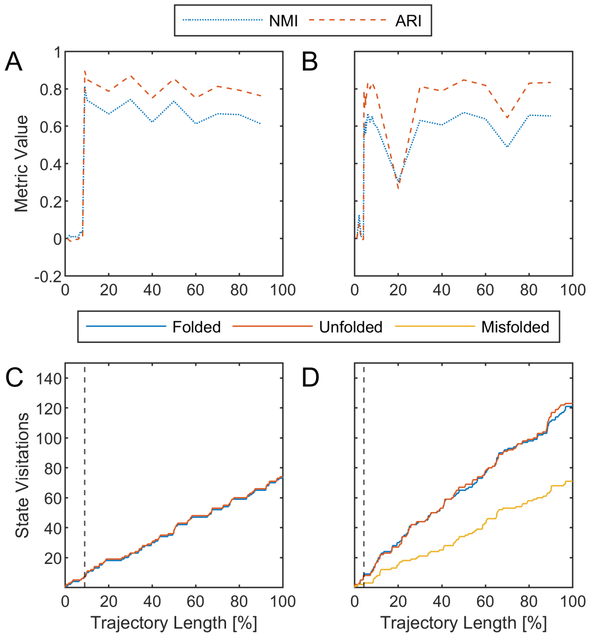

Figure S14: Classification metric values and state visitations versus the CV trajectory length. (A) NMI (dotted blue) and ARI (dashed orange) classification metrics values versus the trajectory length for Chignolin and (B) Trp-Cage. The trajectory length is measured in percentage of the Chignolin (total length: 5348 samples) and the Trp-Cage (total length: 6692 samples) total trajectory length after downsampling. (C) Different protein state visitation counts versus the trajectory length for Chignolin and (D) Trp-Cage, for the folded (blue), unfolded (orange), and misfolded (yellow) states. The dashed vertical black lines correspond to the minimal state visitation counts estimated for both proteins, as described in the text.

References

(1) Zhao, K.; Wulder, M. A.; Hu, T.; Bright, R.; Wu, Q.; Qin, H.; Li, Y.; Toman, E.; Mallick, B.; Zhang, X. et al. Detecting change-point, trend, and seasonality in satellite time series data to track abrupt changes and nonlinear dynamics: A Bayesian ensemble algorithm. *Remote Sen. Environ.* **2019**, *232*, 111181.

(2) Ester, M.; Kriegel, H.-P.; Sander, J.; Xu, X. *Proceedings of the Second International Conference on Knowledge Discovery and Data Mining*; AAAI Press, 1996; p 226–231.

(3) Schubert, E.; Sander, J.; Ester, M.; Kriegel, H. P.; Xu, X. DBSCAN revisited, revisited: why and how you should (still) use DBSCAN. *ACM Transactions on Database Systems (TODS)* **2017**, *42*, 1–21.

(4) Shen, J.; Hao, X.; Liang, Z.; Liu, Y.; Wang, W.; Shao, L. Real-time superpixel segmentation by DBSCAN clustering algorithm. *IEEE transactions on image processing* **2016**, *25*, 5933–5942.

(5) Kellner, D.; Klappstein, J.; Dietmayer, K. Grid-based DBSCAN for clustering extended objects in radar data. 2012 IEEE Intelligent Vehicles Symposium. 2012; pp 365–370.

(6) Edla, D. R.; Jana, P. K. A Prototype-Based Modified DBSCAN for Gene Clustering. *Procedia Technology* **2012**, *6*, 485–492, 2nd International Conference on Communication, Computing & Security [ICCCS-2012].

(7) Saeedi Emadi, H.; Mazinani, S. M. A novel anomaly detection algorithm using DBSCAN and SVM in wireless sensor networks. *Wireless Personal Communications* **2018**, *98*, 2025–2035.

(8) Khan, K.; Rehman, S. U.; Aziz, K.; Fong, S.; Sarasvady, S. DBSCAN: Past, present and future. The fifth international conference on the applications of digital information and web technologies (ICADIWT 2014). 2014; pp 232–238.

(9) Sander, J.; Ester, M.; Kriegel, H.-P.; Xu, X. Density-based clustering in spatial databases: The algorithm gdbscan and its applications. *Data mining and knowledge discovery* **1998**, *2*, 169–194.

(10) Kaplan, D. Knee Point. `https://www.mathworks.com/matlabcentral/fileexchange/35094-knee-point`, 2024; [Online; accessed January 22, 2024].

(11) Chen, Y.; Tang, S.; Zhou, L.; Wang, C.; Du, J.; Wang, T.; Pei, S. Decentralized Clustering by Finding Loose and Distributed Density Cores. *Information Sciences* **2018**, *433-434*, 510–526.

(12) Kuhn, H. W. The Hungarian method for the assignment problem. *Naval research logistics quarterly* **1955**, *2*, 83–97.

(13) Cohen, I.; Huang, Y.; Chen, J.; Benesty, J.; Benesty, J.; Chen, J.; Huang, Y.; Cohen, I. Pearson correlation coefficient. *Noise reduction in speech processing* **2009**, 1–4.
